# Comprehensive Bioinformatics Analysis of Lipopolysaccharide-Induced Altered Autophagy in Acute Lung Injury and Construction of Underlying Competing Endogenous RNA Regulatory Mechanism

**DOI:** 10.1155/2021/6831770

**Published:** 2021-10-21

**Authors:** Jian-Yu Liu, Ying-Xiao Jiang, Meng-Yu Zhang, Chen Huo, Yi-Can Yang, Xiu-Li Ji, Yi-Qing Qu

**Affiliations:** ^1^Department of Pulmonary and Critical Care Medicine, Qilu Hospital, Cheeloo College of Medicine, Shandong University, Shandong Key Laboratory of Infectious Respiratory Diseases, China; ^2^Department of Pulmonary Disease, Jinan Traditional Chinese Medicine Hospital, China; ^3^Department of Pulmonary and Critical Care Medicine, Qilu Hospital of Shandong University, Shandong Key Laboratory of Infectious Respiratory Disease, China

## Abstract

**Background:**

Acute lung injury (ALI) is a fatal syndrome frequently induced by lipopolysaccharide (LPS) released from the bacterial cell wall. LPS could also trigger autophagy of lung bronchial epithelial cell to relieve the inflammation, while the overwhelming LPS would impair the balance of autophagy consequently inducing serious lung injury.

**Methods:**

We observed the autophagy variation of 16HBE, human bronchial epithelial cell, under exposure to different concentrations of LPS through western blot, immunofluorescence staining, and electron microscopy. Eight strands of 16HBE were divided into two groups upon 1000 ng/ml LPS stimulation or not, which were sent to be sequenced at whole transcriptome. Subsequently, we analyzed the sequencing data in functional enrichment, pathway analysis, and candidate gene selection and constructed a hsa-miR-663b-related competing endogenous RNA (ceRNA) network.

**Results:**

We set a series of concentrations of LPS to stimulate 16HBE and observed the variation of autophagy in related protein expression and autophagosome count. We found that the effective concentration of LPS was 1000 ng/ml at 12 hours of exposure and sequenced the 1000 ng/ml LPS-stimulated 16HBE. As a result, a total of 750 differentially expressed genes (DEGs), 449 differentially expressed lncRNAs (DElncRNAs), 76 differentially expressed circRNAs (DEcircRNAs), and 127 differentially expressed miRNAs (DEmiRNAs) were identified. We constructed the protein-protein interaction (PPI) network to visualize the interaction between DEGs and located 36 genes to comprehend the core discrepancy between LPS-stimulated 16HBE and the negative control group. In combined analysis of differentially expressed RNAs (DERNAs), we analyzed all the targeted relationships of ceRNA in DERNAs and figured hsa-miR-663b as a central mediator in the ceRNA network to play when LPS induced the variation of autophagy in 16HBE.

**Conclusion:**

Our research indicated that the hsa-miR-663b-related ceRNA network may contribute to the key regulatory mechanism in LPS-induced changes of autophagy and ALI.

## 1. Introduction

ALI, an overwhelming inflammatory response within lung and severely impaired function of gas change [1, 2], is a common syndrome with high morbidity and mortality which is induced by endotoxins, complement activation, acid aspiration, and hyperoxia [3]. LPS released from the bacterial cell wall is proved to be a vital incentive for ALI development, which could activate various inflammatory pathways [4–6] and result in imbalance of autophagy [7, 8]. Concerning to researches of lung diseases, LPS could facilitate kinds of cells in lung and disease models of mouse to release proinflammatory factors and change in structures of tissue [4, 6, 9–11].

Autophagy is an evolutionarily conserved catabolic process for degrading intracellular component to maintain cellular homeostasis and accommodate to environmental irritation [12]. Within the impaired response caused by LPS, autophagy plays a predominant role in regulatory mechanism and reveals interaction with inflammation-related signaling pathways. Zeng et al. constructed LPS-induced mouse model of ALI and demonstrated that 4-phenyl butyric acid (4-PBA) could reduce the release of the proinflammatory mediators by inhibiting nuclear factor kappa-B (NF-*κ*B) pathway and decrease autophagy flux in ALI mouse via activation of AKT serine/threonine kinase 1 (AKT)/mammalian target of rapamycin (mTOR) signaling pathway [8]. However, their further studies showed that 3-methyladenine (3-MA), a classic inhibitor of autophagy, increased the endoplasmic reticulum (ER) stress and exacerbated cytotoxicity induced by LPS, which indicated autophagy display a protective effect on the progression of ALI. In mice and MH-S cells, Zhao et al. also demonstrated there is severely impaired function of autophagy in LPS-induced ALI model [13]. The levels of cytokines, including interleukin- (IL-) 6, IL-1*β*, and tumor necrosis factor alpha (TNF-*α*), are decreased by application of rapamycin, and cell viability is improved with rescued function of autophagy. Similarly, Zhang et al. investigated the regulatory mechanism of autophagy in LPS-induced pulmonary damage. They elaborated that, in the human pulmonary microvascular endothelial cells (HPMVECs), LPS would lead to higher permeability, lower vitality, and increased lactate dehydrogenase (LDH) release rate, and these changes would be aggravated when inhibiting autophagy. In animal experiments, LPS caused severe pulmonary damage, including hemorrhage, leukocyte infiltration and edema in lung tissue, and high level of proinflammatory cytokines, which was also aggravated by inhibition of autophagy [7]. To further investigate the underlying regulatory mechanism between autophagy and ALI, Nosaka et al. construct the mice with myeloid-specific deletion of the autophagic protein ATG16L1 (*Atg16l1*^fl/fl^*LysM*^Cre^). They found that the mice suffered hypoxemia and increased lung permeability with significantly higher level of IL-1*β*, which indicated that autophagy exerted a protective role in suppressing inflammasome activation and production of IL-1*β* [14]. Except for the researches for discussing autophagy in ALI, autophagy also exerts a crucial role for maintaining homeostasis in other LPS-induced and inflammatory disease model. Kong et al. revealed that LPS decreases the numbers of hepatic autophagosome on the exposure of alcohol, and rapamycin could reverse the ethyl alcohol (EtOH)-LPS-induced liver injury via interaction of Toll-like receptor (TLR4)/lymphocyte antigen 96 (MD2) signaling complex [15]. LPS stimulates Leydig cell in accumulation of oxidative stress and causes turbulence of mitochondria, which is the important influential factor involved in the steroidogenic impairment of Leydig cells [16–18]. Li et al.'s research showed that adrenomedullin (ADM) promotes autophagy of Leydig cells to play a protective role of pyroptosis and cell biological functions in response to the exposure of LPS [19]. They compared the ADM with rapamycin and found the similar effect on the phosphorylation of adenosine 5′-monophosphate- (AMP-) activated protein kinase (AMPK)/mTOR signaling pathway, and the combination of ADM and rapamycin exerts synergistic effect to lessen LPS-induced injury of Leydig cells. The protective effects of autophagy in inflammatory diseases are verified in various cell lines, such as RAW264.7 macrophages [20, 21], BV2 microglial cells [22], adipose-derived stem cells (ADSCs) [23], Caco-2 and HT-29 colonic adenoma cells [24], hepatic stellate cells [25], and microglia [26].

ceRNA can bridge the interplay with autophagy and phenotypes of other diseases, especially in cancer and age-related diseases [27, 28]. In common, ceRNA include partial long noncoding RNAs (lncRNAs) and circular RNAs (circRNAs), which are noncoding RNAs (ncRNAs). MicroRNAs (miRNAs) are another kind of ncRNA and can regulate different physiological and pathological processes by targeting messenger RNA (mRNA). lncRNA and circRNA could competitively sponge with targeted miRNA to exert function of ceRNA to release the targeted mRNA [29, 30]. ceRNA can participate in regulating initiation to maturation progresses of autophagy, which modulate autophagy phagophore initiation through upregulating expression of mTOR, unc-51-like autophagy-activating kinase 1 (ULK1), autophagy-related (ATG) 14L, and Beclin-1. ceRNA also can upregulate ATG3, ATG4, ATG5, ATG7, and ATG12 to influence autophagy phagophore elongation [27]. In acute promyelocytic leukemia (APL)-ascites mouse model and APL cell lines, lncRNA HOTAIRM1 facilitates formation of autophagosome to degrade oncoprotein PML nuclear body scaffold (PML)-retinoic acid receptor alpha (RARA) via targeting miR-20a [31]. In contrast, HOX transcript antisense RNA (HOTAIR) could sponge miR-93 to upregulate ATG12 expression in colorectal cancer cells, which is reported to induce autophagy and decrease radiosensitivity [32]. lncRNA PTENP1 can promote autophagy, as a sponge of miR-17, miR-19b, and miR-20a, by targeting ULK1, ATG7, p62, phosphatase and tensin homolog (PTEN), and PH domain and leucine-rich repeat protein phosphatase 1 (PHLPP), which could promote progression of hepatocellular carcinoma [33, 34]. Concerning to age-related diseases (ARDs), lncRNAs trigger cellular senescence and the senescence-associated secretory phenotypes (SASPs) [28], which are suggested as the two major contributors to inflammation [35]. NIFK antisense RNA 1 (NIFK-AS1) and colon cancer-associated transcript 1 (CCAT1) sponge miR-148a and miR-146a, respectively, to suppress macrophage M2 polarization and malignant behaviors [36, 37]. lncRNA myocardial infarction-associated transcript (MITA), growth arrest-specific 5 (GAS5), HOTAIR, and urothelial cancer-associated 1 (UCA1) can competitively bind miRNA to promote macrophage M1 polarization, which induces upregulated level of ROS, proinflammatory cytokines, and matrix metalloproteinase [38–41]. Among the mutual transformation of macrophages, M1 polarization is proinflammatory while M2 polarization is anti-inflammatory [42]. As for the regulatory mechanism between autophagy and inflammation, ceRNA also play a crucial role in targeting autophagy to influence the progression of inflammation. In vascular endothelial cells (VECs), Huang et al. found that TGFB2 overlapping transcript 1 (TGFB2-OT1), a lncRNA derived from TGFB2, can regulate autophagy [43]. They demonstrated that LPS significantly upregulated the level of TGFB2-OT1 and further use of mRNA chip assay revealed that miR3960, miR4488, and miR4459 are targets of TGFB2-OT1. TGFB2-OT1 sponged miR4488, miR4459, and miR3960 to regulate N-acetyltransferase 8-like (NAT8L), La ribonucleoprotein 1, translational regulator (LARP1), and ceramide synthase 1 (CERS1), which could affect mitochondrial functions by participating in autophagy and induce production of IL-6, IL-8, and IL-1*β*. Our aim is to investigate the regulatory mechanism of ceRNA between autophagy and LPS-induced ALI and find out the key mediators among the ceRNA network.

## 2. Material and Methods

### 2.1. Cell Culture, Antibodies, and Reagents

Immortalized bronchial epithelial cell line 16HBE cells were obtained from Procell Technology (Wuhan, China). They were cultured in RPMI 1640 (Gibco) at 37°C with 5% CO_2_ and cocultured with 10% foetal bovine serum (Biological Industries). According to instruction, we dissolve the LPS powder into different concentrations and stimulate 16HBE for 12 hours.

Antibodies used were rabbit anti-LC3B (Cell Signaling Technology, 3868), rabbit anti-SQSTM1/p62 (Cell Signaling Technology, 8025), and mouse anti-GAPDH (Beyotime, AF5009). Lipopolysaccharide (Sigma-Aldrich, L4391), DAPI (Solarbio, C0065), and RAPA (Sigma-Aldrich, 553210) were used in this study.

### 2.2. Sample Collection and Preparation

RNA degradation was operated on 1.5% agarose gels. RNA integrity was assessed using the RNA Nano 6000 Assay Kit (Agilent Technologies, CA, USA). RNA concentration and purity were measured using the NanoDrop 2000 Spectrophotometer (Thermo Fisher Scientific, Wilmington, DE).

The rRNA removal used the Ribo-Zero rRNA Removal Kit (Epicentre, Madison, WI, USA) with 1.5 *μ*g RNA per sample. Sequencing libraries were generated using NEBNext^R^ Ultra™ Directional RNA Library Prep Kit for Illumina^R^ (NEB, USA) following the manufacturer's recommendations. Finally, PCR products were purified (AMPure XP system) and library quality was assessed on the Agilent Bioanalyzer 2100 and qPCR.

RNA sample preparations consumed 2.5 ng RNA per sample as input material. Sequencing libraries were generated using NEBNext^R^ Ultra™ small RNA Sample Library Prep Kit for Illumina^R^ (NEB, USA) following the manufacturer's recommendations. PCR products were purified, and library quality was assessed as mentioned above.

After cluster generation using TruSeq PE Cluster Kit v3-cBot-HS (Illumina), the library preparations were sequenced on an Illumina platform and reads were generated.

### 2.3. Quantification of Gene Expression Levels and Significant Differentially Expressed RNAs Screening

The levels of gene expression were estimated by fragments per kilobase of transcript per million fragments mapped, i.e., FPKM. The formula is shown as follows:

(1) $$\begin{array}{c} \text{FPKM}=\frac{\text{cDNA fragments}}{\text{Mapped fragments }\left(\text{millions}\right)\times \text{Transcript length}\left(\text{kb}\right)}. \end{array}$$

Differential expression analysis was performed using the DESeq R package. The resulting *P* values were adjusted using the Benjamini and Hochberg approach for controlling the false discovery rate. Genes with an adjusted *P* value < 0.01 and absolute value of log_2_ (fold change) > 1 found by DESeq were assigned as differentially expressed. The DERNAs, including DElncRNAs, DEmiRNAs, DEcircRNAs, and DEGs, were identified by the limma package in R which was performed to identify. The ggplot2 R package was used to generate volcano plot. LogFC > 0 indicated that genes were downregulated; in contrast, genes were upregulated with logFC < 0. The significant differentially expressed RNAs were defined as*P* < 0.05and∣logFC | >1.5.

### 2.4. The Functional Enrichment and Pathway Analysis

Gene Ontology (GO) enrichment analysis of the DEGs was implemented by the clusterProfiler R packages. Enrichment analysis uses hypergeometric testing to find GO entries that are significantly enriched compared to the entire genome background. Gene set enrichment analysis (GSEA) can also be analyzed by clusterProfiler.

Kyoto Encyclopedia of Genes and Genomes (KEGG) is a database resource for comprehending the advanced functions and biosystem in molecular level, which could make relevance between sequenced genomes and systemic functions of cell, species, and ecosystem. We found the significantly enriched KEGG pathway of DEGs via clusterProfiler R packages compared to the entire genome background.

### 2.5. Candidate Gene Selection

We used Search Tool for the Retrieval of Interacting Genes (STRING) (http://string-db.org/) to figure out the interaction relationship for these DEGs with confidence score > 0.4 [44]. Then, PPI network of DEGs was visualized by Cytoscape [45]. According to the default algorithm of the cytoHubba plugin, ranking nodes indicated the importance of networks [46], which help us locate hub genes. The Molecular Complex Detection (MCODE) plugin was performed to select linked genes with degree cutoff = 2 and *k* − core = 2.

### 2.6. Construction of a hsa-miR-663b-Related ceRNA Network and the Combination Pathway Analysis of Host Gene

Our research obtained the candidate ceRNA pairs via miRNA-targeted relationship and satisfy the following conditions: (a) the number of identical miRNAs between ceRNA pairs should be greater than 5 and (b) *P* value < 0.01 and adjusted FDR < 0.05. We found that hsa-miR-663b was the key regulator between these selected ceRNA pairs, which was related to many ceRNA pairs. The combination pathway analysis of host gene used the classical algorithm PageRank in the random walk model to obtain the score of all nodes in network. Ranging these scores and selecting the top 10 percent as crucial nodes, these nodes were analyzed in pathway enrichment and were mapped into the top 5 enriched pathways. Then, we used igraph R package to visualize these relationships.

### 2.7. Western Blot Analysis

16HBE were lysed in RIPA buffer (Beyotime, P0013), which were grown on 6-well culture plates. Then, equal amounts of extracted proteins (10-30 *μ*g) were separated in 10-15% SDS-PAGE and transferred to the polyvinylidene difluoride membrane (Immobilon-P Transfer Membranes, IPVH00010, 0.45 *μ*m) by electrophoresis. The membranes were incubated with specific primary antibody for 1 h at room temperature and afterwards were incubated by anti-mouse IgG, HRP-linked secondary antibody (Cell Signaling Technology, 7076) or anti-rabbit IgG, HRP-linked secondary antibody (Cell Signaling Technology, 7074). When finished the above footsteps, the membranes were detected by chemiluminescence detection (Tannon 4800). The grayscale of the bands was quantified using ImageJ (Version 1.58j8), and the data were normalized to the GAPDH loading controls.

### 2.8. Electron Microscopy (EM)

16HBE were stimulated with different concentrations of LPS for 12 h. After treatment, 16HBE were fixed with 2% glutaraldehyde/0.1 M phosphate buffer (pH 7.4) and in 1% osmium tetroxide/0.1 M phosphate buffer (pH 7.4) and dehydrated with a graded series of ethanol. Then, we embedded these fixed 16HBE into epoxy resin. Ultrathin sections were stained with lead citrate and uranyl acetate. The sections were observed with the Hitachi H-7500 transmission electron microscope (Hitachi, Tokyo, Japan). For quantitative evaluation of autophagosomes in 16HBE, 16 image fields (8 of 1500x and 5000x, respectively) were selected for each sample.

### 2.9. Immunofluorescence Staining

16HBE cells grown on 36-well culture slides were fixed with 4% paraformaldehyde for 15 min and were permeated with 0.03% Triton X-100 (Solarbio, T8200) for 20 min. 5% BSA (BioFroxx, 4240) was used to block cells for 30 min; then, the primary antibodies were incubated for 12-18 hours and secondary antibodies were incubated for 1 hour at room temperature. Fluorescence microscopy (Olympus, IX81, Japan) analysis of LC3B and DAPI staining were performed in 16HBE.

### 2.10. Statistical Analysis

Statistical analysis was performed using GraphPad Prism 7.00 and R 4.0.5 software. Date are shown as the average (±SEM) taken from at least 3 independent experiments. Parametric data between 2 different groups were compared by the Student *t*-test. The variance for multiple comparisons was determined by one-way analysis. Significance was defined as *P* < 0.05. The statistical methods of bioinformatics analysis were detailed as mentioned previously.

## 3. Results

### 3.1. LPS-Induced Autophagy in Lung Epithelial Cells

**Figure 1:**
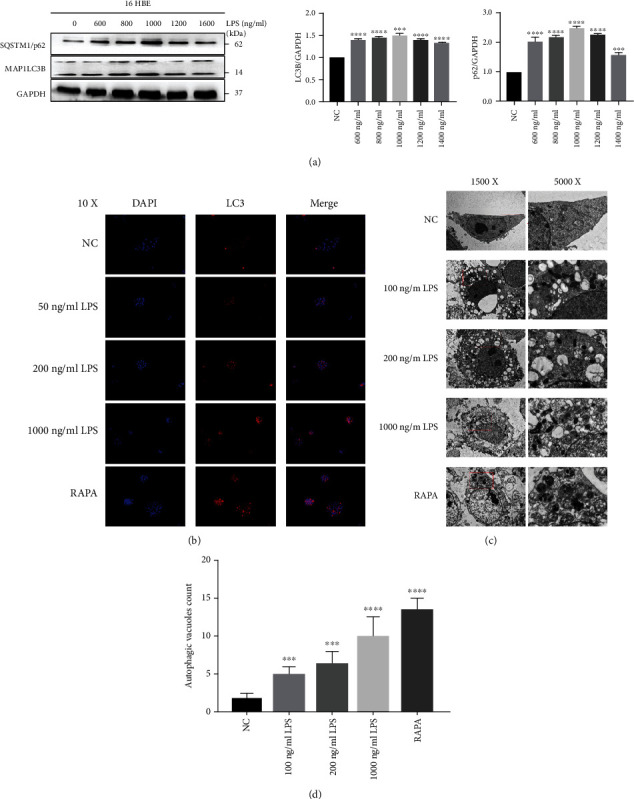
LPS induces autophagy in 16HBE. (a) WB using anti-MAP1LC3B, anti-SQSTM1/p62, and anti-GAPDH. 16HBE were treated with different concentrations of LPS (0-1600 ng/ml) and RAPA (50 nM) for 12 h, and protein samples were collected. Shown is a representative experiment of 2 showing similar results. The middle panel is the average (±SEM) of the relative increase in LC3B normalized to GAPDH and the right panel is the average (±SEM) of the relative increase in SQSTM1/p62 normalized to GAPDH. ^∗∗∗^*P* < 0.001 and ^∗∗∗∗^*P* < 0.0001. (b) Fluorescence microscopy detection (10x) of DAPI (left panels) and LC3 (right panels). 16HBE were treated with different concentrations of LPS (0-1600 ng/ml) for 12 h. (c) Electron microscopy detection of autophagosome in 16HBE with different concentrations of LPS (0-1600 ng/ml) and RAPA (50 nM). (d) Shown in this panel is average (±SEM) of autophagosome counts taken from 6 image fields (1500x) for each sample. ^∗∗∗^*P* < 0.001 and ^∗∗∗∗^*P* < 0.0001.

To explore the alteration of autophagy when 16HBE suffered from the stimulation of LPS, we set different concentrations of LPS and observed the changes of autophagy via western blotting, immunofluorescence staining, and electron microscopy (Figure 1). To preliminarily confirm the variation trend of autophagy in LPS-induced 16HBE cell lines, we detected the expression of microtubule-associated protein 1 light chain 3 beta (MAP1LC3B) and sequestosome 1 (SQSTM1/p62). The expression of MAP1LC3B-I and SQSTM1/p62 appeared similar trend which rose firstly along with the higher concentration of LPS and reached climax in 1000 ng/ml of LPS. Nevertheless, the expression of them declined with unceasingly increasing concentration of LPS. The expression of MAP1LC3B-II was apparently upregulated compared to the negative control group, whereas it showed no obvious discrepancy between the LPS-induced groups (Figure 1(a)). In immunofluorescence, we labeled autophagosomes and nuclei with LC3-II punctate dots and DAPI which emitted red and blue colors, respectively (Figure 1(b)). We set the NC group and RAPA group as the negative and positive control groups and found that there were more LC3B punctate dots in the 1000 ng/ml LPS-stimulated group. Subsequently, we verified the identical phenomenon through electron microscopy (EM) evaluation (Figure 1(c)). LPS significantly increased the number of autophagic vacuoles, and the change was mostly obvious in the 1000 ng/ml LPS-stimulated group (Figure 1(d)). Under observations, most vacuoles were late/degradative autophagic vacuoles/autolysosomes (AVd or AVl) in our research which typically had only one limiting membrane and contained electron-dense cytoplasmic material.

### 3.2. Differentially Expressed Genes in LPS-Stimulated Lung Epithelial Cells

**Figure 2:**
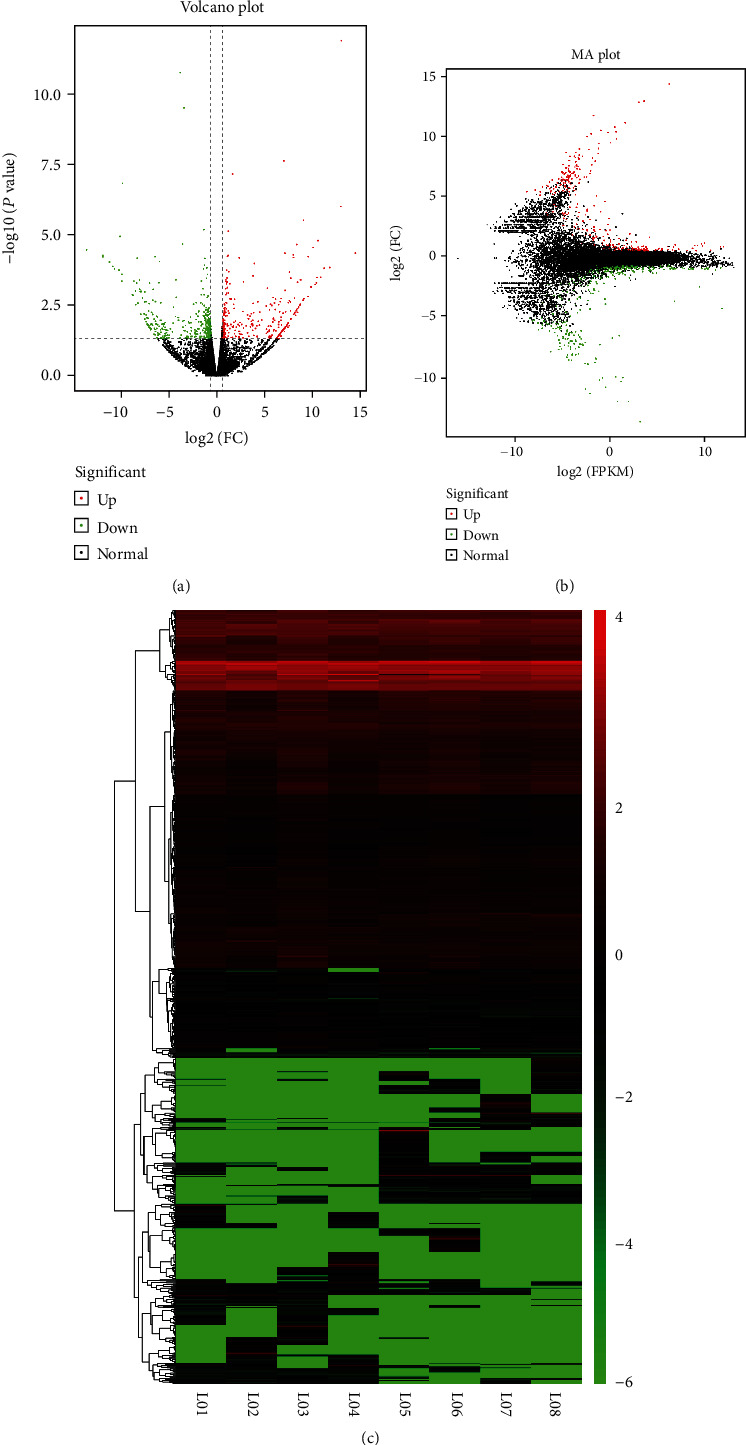
Comprehensive analysis of differentially expressed genes. (a) The volcano plot of differentially expressed genes. (b) The MA plot of differentially expressed genes. (c) The heat map of differentially expressed genes.

We constructed 8 strains of 16HBE into two groups as the negative control group and LPS-stimulated group, and they all included 4 strains of the isogenous 16HBE. The stimulative concentration of LPS was 1000 ng/ml, and stimulative time was 12 hours. Then, we made the whole transcriptome sequencing analysis in these cells to figure out the alterations in 16HBE after LPS-related stimulation. We obtained 750 DEGs and constructed volcano plot to display the distribution of DEGs in the dimensions of -log_10_ (*P* value) and log_2_ (FC). On the basis of *P* < 0.05 and ∣FC | >1.5, 312 genes were upregulated and 438 were downregulated as shown in Figure 2(a). To intuitively understand the overall distribution of expression levels and fold changes, we also demonstrate the results in MA plot (Figure 2(b)). We made hierarchical clustering analysis and display it in heat map (Figure 2(c)).

### 3.3. Differentially Expressed Noncoding RNAs in LPS-Stimulated Epithelial Cells

**Figure 3:**
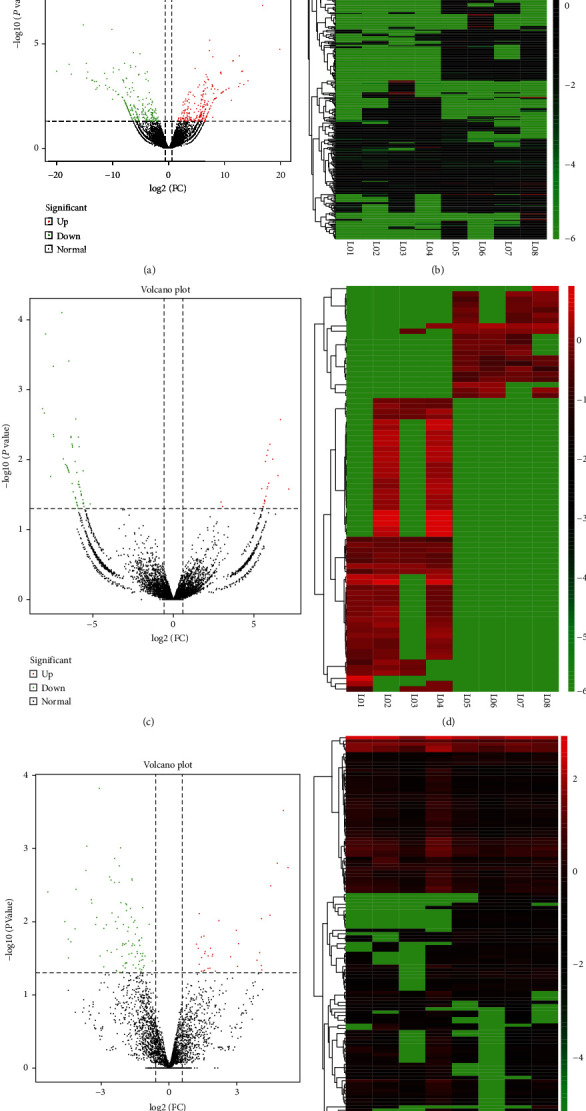
Comprehensive analysis of differentially expressed RNAs. (a) The volcano plot of differentially expressed lncRNAs. (b) The heat map of differentially expressed lncRNAs. (c) The volcano plot of differentially expressed circRNAs. (d) The heat map of differentially expressed circRNAs. (e) The volcano plot of differentially expressed miRNAs. (f) The heat map of differentially expressed miRNAs.

Except for sequencing in mRNAs, we also sequenced noncoding RNAs (ncRNA) including lncRNAs, circRNAs, and miRNAs. The volcano plots and heat maps of differentially expressed noncoding RNAs are shown in Figures 3(a)–3(f). Similarly, we set the screen criteria as *P* < 0.05 and ∣FC | >1.5. We found more differentially expressed ones in lncRNAs, which contained 230 upregulated and 219 downregulated lncRNA. circRNAs only revealed 76 differentially expressed ones in our study (Table 1). These heat maps demonstrated that there were existing variations between the negative control groups and LPS-stimulated groups which furtherly prompted underlying ceRNA regulatory network in 16HBE when suffering exposure of LPS (Figure 3).

### 3.4. Enrichment Analysis of Differentially Expressed Genes and ncRNAs in LPS-Stimulated Epithelial Cells

**Figure 4:**
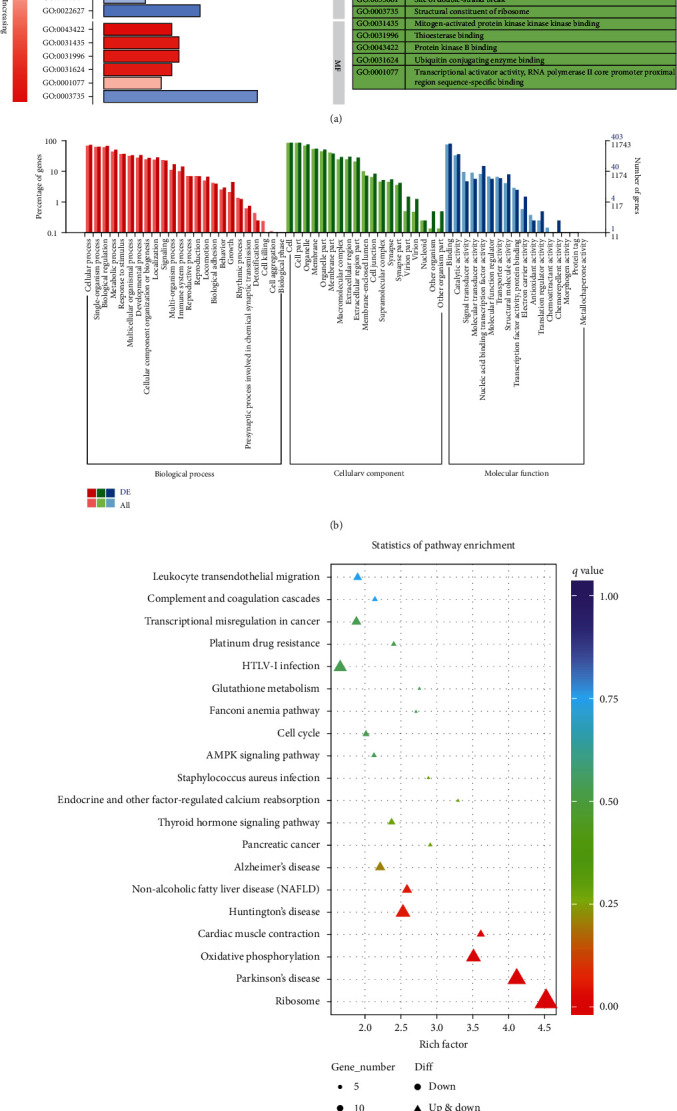
Functional enrichment analysis of differentially expressed genes in LPS-stimulated 16HBE. (a) Gene Ontology terms of differentially expressed genes in LPS-stimulated 16HBE. The right panel is the annotations of ID of GO terms. (b) The discrepancy of GO terms between differentially expressed genes and whole genes. (c) The KEGG enrichment analysis of differentially expressed genes in LPS-stimulated 16HBE via clusterProfiler R package. The horizontal ordinate is enrichment factor which shows the ratio of differentially expressed genes enriched in the pathway to whole genes enriched in the pathway. The vertical ordinate is -log_10_ (*Q*-value).

To investigate functions of the differentially expressed genes and ncRNAs, we performed GO and KEGG using clusterProfiler of R. Enriched terms with *P* < 0.05 were displayed (Figure 4 and S1). The genes were mainly enriched in viral transcription, nuclear-transcribed mRNA catabolic process, and SRP-dependent cotranslational protein targeting to the membrane in biological process; CD40 receptor complex and cytoplasmic side of the plasma membrane in cellular component; structural constituent of ribosome and mitogen-activated protein kinase (MAPK) kinase kinase binding in molecular function (Figures 4(a) and 4(b)); and ribosome, Parkinson's disease, and oxidative phosphorylation in KEGG (Figure 4(c)). The cis-targeted genes of lncRNAs were enriched in blood coagulation and Fc-epsilon receptor signaling pathway in biological process (Figure S1A), the lysosomal membrane and endosome membrane in cellular component (Figure S1B), identical protein binding and histone deacetylase binding in molecular function (Figure S1C), and HTLV-I infection and Herpes simplex infection in KEGG (Figure S1D). The trans-targeted genes of lncRNAs were enriched in nuclear-transcribed mRNA catabolic process and translation initiation in biological process (Figure S1E), CD40 receptor complex in cellular component (Figure S1F), structural constituent of ribosome and ubiquitin-conjugating enzyme binding in molecular function (Figure S1G), and ribosome, oxidative phosphorylation, and Parkinson's disease in KEGG (Figure S1H). The circRNAs were mainly enriched in positive regulation of cell migration in biological process (Figure S1I), while there was no predominant result in KEGG under limitation of *P* < 0.05. The miRNA was mainly enriched in dendrite and neuron projection in cellular component (Figure S1J), protein kinase binding in molecular function (Figure S1K), and pathway in cancer, endocytosis, and the MAPK signaling pathway in KEGG (Figure S1L).

### 3.5. Construction of the PPI Network and Screening of Modules and Hub Genes

**Figure 5:**
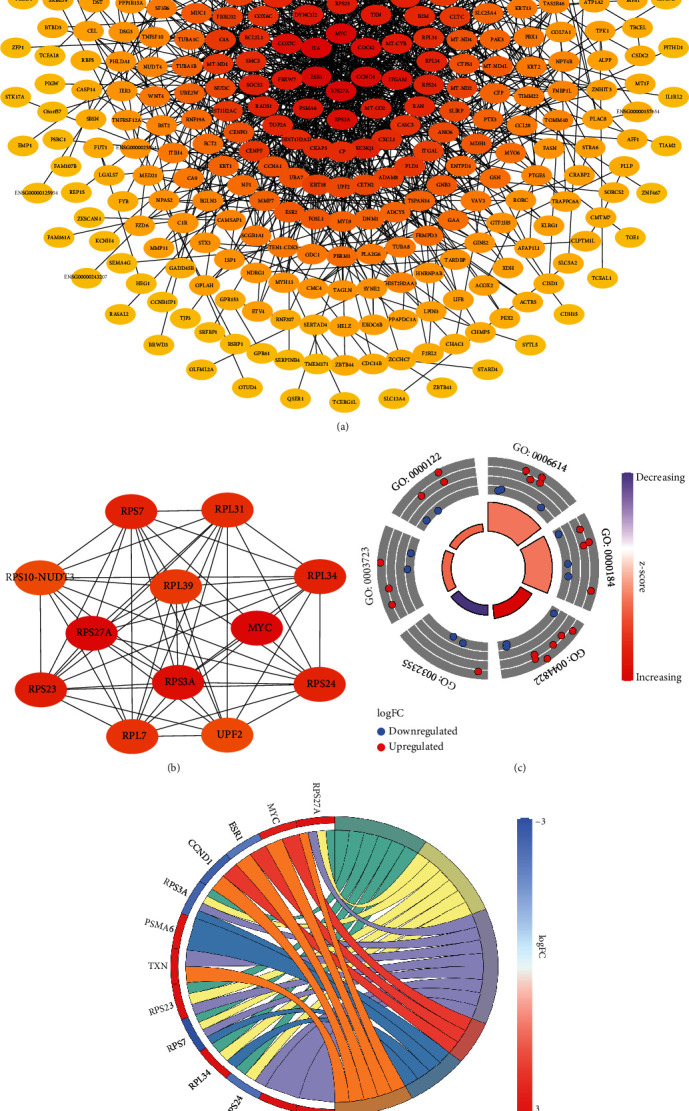
Screening the key genes and further GO analysis from PPI network. (a) The PPI network constructed via STRING. (b) Hub genes selected using cytoHubba. (c) The outer circle represents the expression (logFC) of 36 differentially expressed genes in each enriched GO (Gene Ontology) terms. The inner circle indicates the significance of GO terms (log_10_-adjusted *P* values). Red points indicate upregulated gene, and blue points indicate downregulated gene. (d) The circle indicates the correlation between hub genes and their Gene Ontology terms.

To furtherly figure out the significant proteins and biological modules which played a vital role in the LPS-induced ALI, we constructed PPI network via utilizing STRING and visualized the differentially expressed genes through Cytoscape software and cytoHubba (Figure 5(a)). Then, we located top 12 hub genes (UPF2, MYC, RPL34, RPL39, RPS3A, RPS23, RPS7, RPL7, RPL31, RPS24, RPS27A, RPS10-NUDT3) by calculating maximal clique centrality (MCC) which was the top 1 module and revealed the most densely connected region (Figure 5(b)). We selected 36 genes (IL6, RPS27A, MYC, ESR1, CCND1, CDC42, COX7C, RPS3A, ITGAM, PSMA6, FBXW7, DYNC1I2, TXN, MT-CYB, RPS23, MT-CO2, RAD51, SOCS3, HINT1, RPS7, RPL34, RPS24, KCNQ1, TOP2A, NDUFA5, MT-CO3, B2M, RAN, CKAP5, HIST1H2AD, SMC3, BCL2L1, RAB11A, RPL31, CXCL1, CP) via degree analysis (degree value ≥ 15) and made functional annotation of these 36 genes by M-code which illustrated the vital functions in 6 GO terms (*P* < 0.05). The results are showed in Table 2 and contained RNA binding, nuclear-transcribed mRNA catabolic process, nonsense-mediated decay, poly(A) RNA binding, response to estradiol, SRP-dependent cotranslational protein targeting to the membrane, and negative regulation of transcription from RNA polymerase II promoter (Figures 5(c) and 5(d)).

### 3.6. Combined Analysis and Construction of hsa-miR-663b-Related ceRNA Network

**Figure 6:**
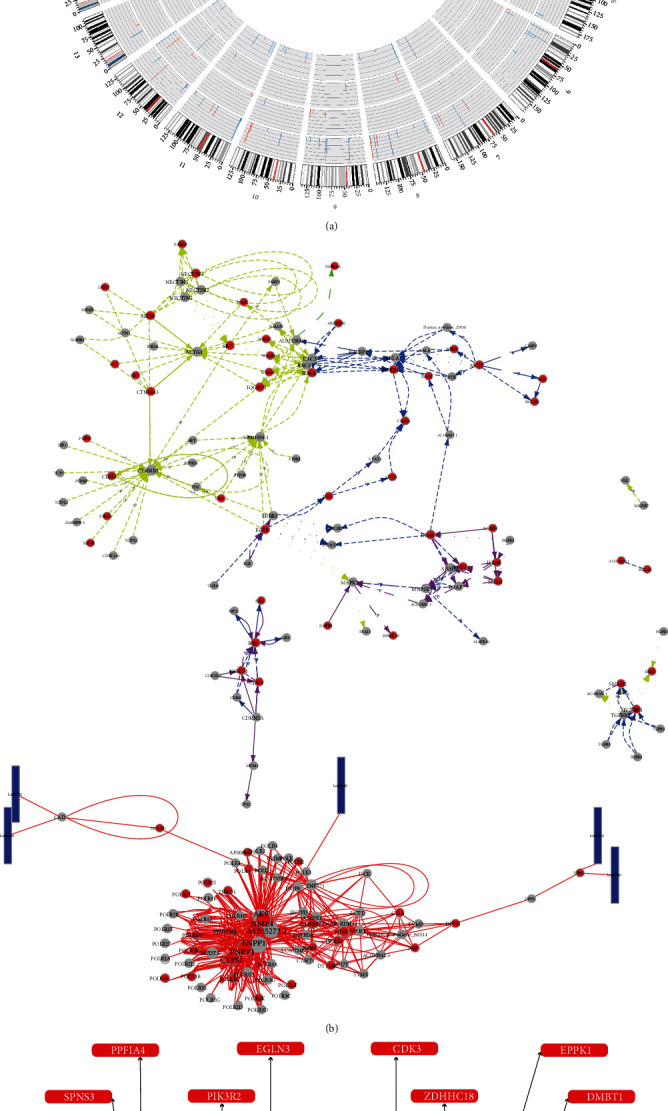
Combined analysis of whole transcriptome and construction of hsa-miR-663b-related ceRNA network. (a) The Circos plot of differentially expressed RNAs. The outermost circle represents chromosome information, then followed by mRNA, lncRNA, circRNA, and miRNA. Red bars indicate upregulated gene, and blue bars indicate downregulated gene. The height of bar represents significance. (b) Pathway enrichment analysis of the crucial RNAs. Dot: gene; rectangle: pathway; red dot: crucial gene; line: interrelation of gene-gene or gene-pathway. (c) Construction of hsa-miR-663b-related ceRNA network.

The differential analysis of whole transcriptome showed differentially expressed mRNA, miRNA, lncRNA, and circRNA of multiple groups, and the overall variation significance were exhibited through Circos (Figure 6(a)). The observation of variations of multiomics data in whole transcriptome was attributed to discover the differential regulatory mechanism. We used the classical algorithm PageRank in the random walk model to obtain the score of all nodes in network which represented the importance in network. We made pathway enrichment analysis of the crucial RNAs in network and then selected the first five pathways with most significant enrichment to illustrate the gene interactions of these pathways via igraph R package (Figure 6(b)). To better predict the upstream regulatory mechanism in the progression of LPS-induced ALI, we constructed a ceRNA network to elaborate the regulatory relationships. According to the whole transcriptome analysis, we took the DElncRNAs or DEcircRNAs as the center and matched corresponding targeted relationships with DEGs and DEmiRNAs to constitute DElncRNA-DEmiRNA-DEG ceRNA pairs (Table 3) and there were no DEcircRNA-DEmiRNA-DEG ceRNA pairs by screening. In these differentially expressed RNA, hsa-miR-663b exerted a central regulatory function (Figure 6(c)). Hence, we presented a hsa-miR-663b-related ceRNA network to explain the regulatory mechanism in LPS-induced ALI, which was conductive for us to furtherly explore the diagnosis and intervention methods of ALI.

## 4. Discussion

ALI was a form of parenchymal lung disease which had various etiologies and usually leaded to fulminant respiratory failure and death. It could develop to acute respiratory distress syndrome (ARDS) and had approximately 33% mortality rate leading to consumption of significant healthcare resources globally [47]. In recent years, it had obtained some achievements in a better comprehension of ALI pathophysiology and proposed some novel measures to pharmacotherapy such as corticosteroid, N-acetylcysteine, statins, surfactants, and antibiotics. Nevertheless, these therapies still had not obviously declined the mortality and morbidity of ALI patient, especially the critical ones [48]. The central feature of ALI was an excessive inflammatory response, but autophagy, as a process for maintaining cellular homeostasis, exerted an ambiguous effect in ALI [8, 49–51]. The activation of mTOR facilitated LPS-induced ALI in mouse, and similarly, 3-MA increased the cytotoxicity of A549 alveolar epithelial cells [8, 49]. The above results proved that inhibition of autophagy generated harmful influence and demonstrated that autophagy plays a protective role in pathogenesis of ALI. While some researchers proposed the opposite view, they discovered that the mechanical ventilation may activate nucleotide-binding oligomerization domain-like receptor containing pyrin domain 3 (NLRP3) inflammasome, which could be suppressed by silencing ATG-5 [50]. Song et al. thought that excessive activating autophagy of alveolar type II epithelial (AT-II) cells is a major cause of ALI and found that microRNA-34a could inhibit the excessive activation of autophagy to relieve LPS-induced ALI via targeting forkhead box O3 (FOXO3) [51]. To investigate the autophagy-related regulatory mechanism in ALI, we constructed LPS-induced ALI model in 16HBE human bronchial epithelial cells.

To observe the alterations of autophagy in 16HBE under LPS exposure, we utilized western blot, immunofluorescent staining, and electron microscopy to evaluate changes of autophagy-related protein expression levels and autophagic vacuole amounts. The expressions of LC3B-I and p62 were obviously upregulated under exposure of LPS and reached the highest levels with the LPS concentration of 1000 ng/ml. Although the expression of LC3B-II was likewise increased compared to the negative control group, it did not appear a significant difference (*P* value > 0.05) under the stimulation of different concentrations of LPS. LC3 were primary Atg8-family homolog examined in mammalian cells and the classic autophagosome marker in autophagy-related researches. LC3-I and LC3-II were the nonlipidated and lipidated forms, respectively [52]. The accumulation of LC3-II is often represented with interruption of the autophagosome-lysosome fusion step [53]. SQSTM1/p62 protein was an index of autophagic degradation, and the lower levels of SQSTM1/p62 usually represented the activation of autophagy [54]. The above results seemingly demonstrated that autophagic flux of 16HBE was interrupted under stimulation of 1000 ng/ml LPS. Under our observation, the amount of autophagic vacuoles was indeed increased with LPS and showed higher levels on the exposure of 1000 ng/ml LPS through immunofluorescent staining and electron microscopy. Generally, the initial autophagic vacuoles, also called autophagosomes, typically had a double membrane [55], whereas there was typically only one limiting membrane in the AVd, and it usually contained electron-dense cytoplasmic and/or organelles at various stages of degradation [56, 57]. In the late digestion process, there were only a few membrane fragments and it was hard to distinguish amphisomes, autolysosomes, and lysosomes [58]. However, the most autophagic vacuoles were single-membrane and the typical double-membrane vacuoles were not significant in our research. These results may demonstrate that when exposed to 1000 ng/ml LPS, the autophagic flux of 16HBE was interrupted in late phage and the degradation of autophagosome may be suppressed by stimulation of LPS.

A total of 750 significant DEGs were identified, which mainly enriched in MAPK kinase kinase binding in molecular function and oxidative phosphorylation pathway. When exposed to LPS, these DEGs may play a crucial regulatory in LPS-induced ALI. Then, we constructed PPI network to visualize the interaction relationship of DEGs and selected 36 core genes to reanalyze for functional enrichment. These genes mainly enriched in 6 different GO terms. In addition, we also analyzed the targeted genes of differentially expressed lncRNA, circRNA, and miRNA for functional enrichment. It was worth to be mentioned that the targeted genes of miRNA mainly enriched in endocytosis and MAPK signaling. Multiple researches had demonstrated that MAPK signaling was one of the upstream regulatory mechanisms of mitophagy. Mangiferin could suppress PTEN-induced kinase 1 (PINK1)-parkin RBR E3 ubiquitin protein ligase (PRKN) mitophagy via protein kinase A (PKA)-MAPK signaling to promote a brown fat-like phenotype in murine C3H10T1/2 mesenchymal stem cells [59]. The sonodynamic therapy could trigger MAPK/p38-PINK1-PRKN-dependent mitophagy, in which the antioxidant suppressed the MAPK/p38 activation and sonotoxicity [60]. What is more, Chen et al. had proved that p38 MAPK could directly phosphorylate PRKN at serine 131 to disturb the protective function of mitophagy [61]. However, if mitophagy is also interrupted along with the suppression of autophagy in LPS-stimulated 16HBE is worth to further exploration.

ceRNA was the research focus in recent years, which was a new transcriptional regulatory mechanism. In combined analysis of several kinds of differentially expressed RNA, we selected the differentially expressed mRNA, lncRNA, circRNA, and miRNA for further analysis of ceRNA relationship. Then, we found hsa-miR-663b as a core molecule in the ceRNA network, which was the target of several regulatory ceRNA. In previous researches, hsa-miR-663b usually was described as a tumor promoter in endometrial cancer, osteosarcoma, bladder cancer, and colorectal cancer [62–65]. However, in an analysis of whole-genome miRNA expression in peripheral total blood samples of patients with acute myocardial infarction (AMI), hsa-miR-663b also showed high sensitivity and specificity for the discrepancy from the control groups, which may reveal hsa-miR-663b could be a significant biomarker for cardiovascular diseases [66]. Interestingly, Ragusa et al. detected the miRNA transcriptome in colorectal cancer with the treatment of MAPK/extracellular signal-regulated kinase (ERK) inhibitors and found that hsa-miR-663b was upregulated in three cell lines and induced downregulation of cyclin D2 (CCND2) [67]. It may hint us that there were underlying regulatory mechanisms between hsa-miR-663b and MAPK signaling. In the next step, we were aiming to explore the expression of hsa-miR-663b in ALI and other lung diseases and then furtherly constructed and verified the hsa-miR-663b-related ceRNA regulatory network.

## 5. Conclusion

Our research constructed a novel hsa-miR-663b-related ceRNA regulatory network in LPS-induced ALI by a whole transcriptome sequencing and comprehensive bioinformatics analysis, which may contribute to the key regulatory mechanism in LPS-induced changes of autophagy and ALI. There may exist an underlying regulatory mechanism between hsa-miR-663b, MAPK signaling, and autophagy/mitophagy. Nevertheless, the expression level of hsa-miR-663b and specific regulatory mechanism of ceRNA in ALI should be furtherly validated by molecular experiments.

## Figures and Tables

**Table 1:** The differentially expressed RNAs in LPS-stimulated 16HBE.

DEG set (L01_L02_L03_L04 vs. L05_L06_L07_L08)	All DEG	Upregulated	Downregulated
lncRNA	449	230	219
circRNA	76	21	55
miRNA	127	34	93

The L01 represents the first strain of 16HBE, and the rest can be known in the same way.

**Table 2:** The top 6 GO terms of 36 differentially expressed genes.

Category	ID	Term	Genes	Adj_*P*val
BP	GO:0006614	SRP-dependent cotranslational protein targeting to membrane	RPS7, RPL31, RPL34, RPS3A, RPS27A, RPS24, RPS23	2.11*E* − 08
BP	GO:0000184	Nuclear-transcribed mRNA catabolic process, nonsense-mediated decay	RPS7, RPL31, RPL34, RPS3A, RPS27A, RPS24, RPS23	8.70*E* − 08
MF	GO:0044822	Poly(A) RNA binding	TOP2A, RPS7, RPL31, RPS3A, TXN, RPS27A, RPS24, RAN, RPS23	9.68*E* − 04
BP	GO:0032355	Response to estradiol	CCND1, MYC, ESR1	0.012963812
MF	GO:0003723	RNA binding	PSMA6, RPS7, RPL31, RPL34, RPS3A	0.019110457
BP	GO:0000122	Negative regulation of transcription from RNA polymerase II promoter	CCND1, MYC, TXN, RPS27A, ESR1	0.046553861

BP: biological process; MF: molecular function.

**Table 3:** The DElncRNA-DEmiRNA-DEG ceRNA pairs.

lncRNA	miRNA	Gene
MSTRG.148111.15	hsa-miR-663b	DAG1
MSTRG.148111.10	hsa-miR-663b	SPNS3
FLJ16779-201	hsa-miR-663b	CHAC1
MSTRG.16479.1	hsa-miR-210-5p	CHAC1
MSTRG.1512.7	novel_miR_1799	PIK3R2
MSTRG.144243.12	hsa-miR-663b	PIK3R2
MSTRG.141087.2	novel_miR_1799; novel_miR_752	PIK3R2
AC103718.1-201	hsa-miR-663b	ZDHHC18
MSTRG.144146.2	hsa-miR-663b	ZDHHC18
AC103718.1-201	hsa-miR-663b	DMBT1
MSTRG.144146.2	hsa-miR-663b	DMBT1
MSTRG.13465.1	hsa-miR-663b	EGLN3
MSTRG.148111.15	hsa-miR-663b	EGLN3
DLEU1-212	novel_miR_1799	CASP14
AL731571.1-201	hsa-miR-663b	CDK3
AC055713.1-201	hsa-miR-663b	PPFIA4
MSTRG.65795.2	hsa-miR-663b	EPPK1

## Data Availability

The raw data used and/or analyzed during the current study are available from the corresponding author on reasonable request.

## Abbreviations

ALI: Acute lung injury
LPS: Lipopolysaccharide
4-PBA: 4-Phenyl butyric acid
NF-*κ*B: Nuclear factor kappa-B
AKT: AKT serine/threonine kinase 1
mTOR: Mammalian target of rapamycin
3-MA: 3-Methyladenine
ER: Endoplasmic reticulum
TNF-*α*: Tumor necrosis factor alpha
IL: Interleukin
LDH: Lactate dehydrogenase
HPMVEC: Human pulmonary microvascular endothelial cell
EtOH: Ethyl alcohol
MD2: Lymphocyte antigen 96
ADM: Adrenomedullin
AMPK: Adenosine 5′-monophosphate- (AMP-) activated protein kinase
ADSC: Adipose-derived stem cell
ceRNA: Competing endogenous RNA
lncRNA: Long noncoding RNA
circRNA: Circular RNA
ncRNA: Noncoding RNA
miRNA: MicroRNA
mRNA: Messenger RNA
ULK1: Unc-51-like autophagy activating kinase 1
ATG: Autophagy-related
APL: Acute promyelocytic leukemia
PML: PML nuclear body scaffold
RARA: Retinoic acid receptor alpha
HOTAIR: HOX transcript antisense RNA
PTEN: Phosphatase and tensin homolog
PHLPP: PH domain and leucine-rich repeat protein phosphatase 1
ARD: Age-related disease
SASP: Senescence-associated secretory phenotype
NIFK-AS1: NIFK antisense RNA 1
CCAT1: Colon cancer-associated transcript 1
MITA: Myocardial infarction-associated transcript
GAS5: Growth arrest-specific 5
UCA1: Urothelial cancer-associated 1
TGFB2-OT1: TGFB2 overlapping transcript 1
VEC: Vascular endothelial cell
CERS1: Ceramide synthase 1
NAT8L: N-Acetyltransferase 8-like
LARP1: La ribonucleoprotein 1, translational regulator
DElncRNA: Differentially expressed lncRNA
DEcircRNA: Differentially expressed circRNA
DEG: Differentially expressed gene
GO: Gene Ontology
GSEA: Gene set enrichment analysis
KEGG: Kyoto Encyclopedia of Genes and Genomes
STRING: Search Tool for the Retrieval of Interacting Genes
PPI: Protein-protein interaction
MCODE: The Molecular Complex Detection
MAP1LC3B: Microtubule-associated protein 1 light chain 3 beta
SQSTM1/p62: Sequestosome 1
EM: Electron microscopy
AVd or AVl: Autophagic vacuoles/autolysosomes
MAPK: Mitogen-activated protein kinase
MCC: Maximal clique centrality
ARDS: Acute respiratory distress syndrome
NLRP3: Nucleotide-binding oligomerization domain-like receptor containing pyrin domain 3
FOXO3: Forkhead box O3
PINK1: PTEN-induced kinase 1
PRKN: Parkin RBR E3 ubiquitin protein ligase
PKA: Protein kinase A
AMI: Acute myocardial infarction
ERK: Extracellular signal-regulated kinase
CCND2: Cyclin D2.

## Supplementary Materials

Supplementary Materials Figure S1A: Gene Ontology terms of cis-targeted genes of differentially expressed lncRNAs in LPS-stimulated 16HBE in biological process. Figure S1B: Gene Ontology terms of cis-targeted genes of differentially expressed lncRNAs in LPS-stimulated 16HBE in cellular component. Figure S1C: Gene Ontology terms of cis-targeted genes of differentially expressed lncRNAs in LPS-stimulated 16HBE in molecular function. Figure S1D: the KEGG enrichment analysis of cis-targeted genes of differentially expressed lncRNAs in LPS-stimulated 16HBE. Figure S1E: Gene Ontology terms of trans-targeted genes of differentially expressed lncRNAs in LPS-stimulated 16HBE in biological process. Figure S1F: Gene Ontology terms of trans-targeted genes of differentially expressed lncRNAs in LPS-stimulated 16HBE in cellular component. Figure S1G: Gene Ontology terms of trans-targeted genes of differentially expressed lncRNAs in LPS-stimulated 16HBE in molecular function. Figure S1H: the KEGG enrichment analysis of trans-targeted genes of differentially expressed lncRNAs in LPS-stimulated 16HBE. Figure S1I: Gene Ontology terms of differentially expressed circRNAs in LPS-stimulated 16HBE in biological process. Figure S1J: Gene Ontology terms of differentially expressed miRNA in LPS-stimulated 16HBE in cellular component. Figure S1K: Gene Ontology terms of differentially expressed miRNA in LPS-stimulated 16HBE in molecular function. Figure S1L: the KEGG enrichment analysis of differentially expressed miRNA in LPS-stimulated 16HBE.
