# Supplementary figures and images for: Comprehensive Bioinformatics Analysis of Lipopolysaccharide-Induced Altered Autophagy in Acute Lung Injury and Construction of Underlying Competing Endogenous RNA Regulatory Mechanism
Source: Biomed Res Int. 2021 Oct 21;2021:6831770. doi: 10.1155/2021/6831770 (PMC8553468; doi:10.1155/2021/6831770)

# Biological\_Process

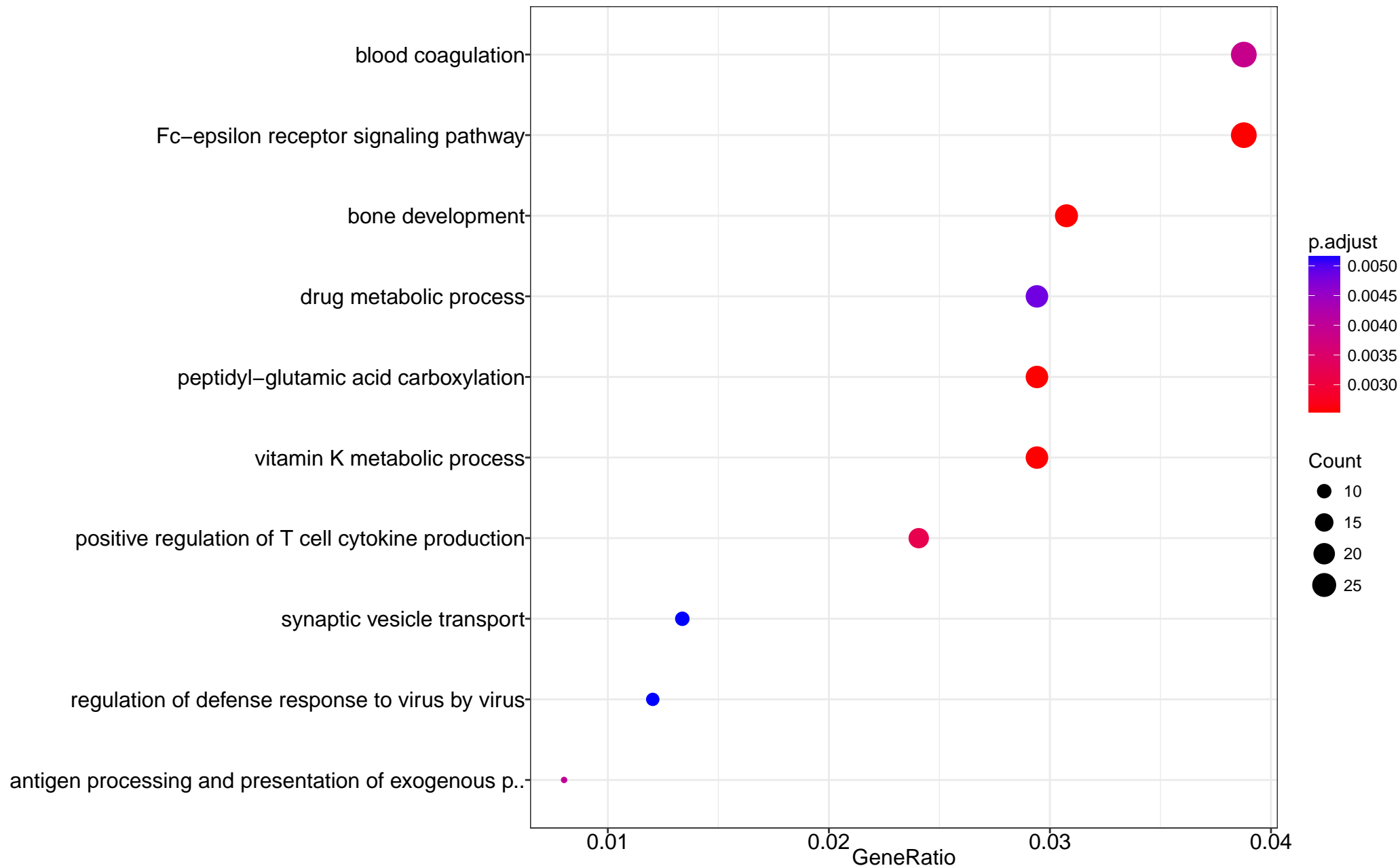

Supplement: Supplementary Materials — Figure S1A: Gene Ontology terms of cis-targeted genes of differentially expressed lncRNAs in LPS-stimulated 16HBE in biological process. Figure S1B: Gene Ontology terms of cis-targeted genes of differentially expressed lncRNAs in LPS-stimulated 16HBE in cellular component. Figure S1C: Gene Ontology terms of cis-targeted genes of differentially expressed lncRNAs in LPS-stimulated 16HBE in molecular function. Figure S1D: the KEGG enrichment analysis of cis-targeted genes of differentially expressed lncRNAs in LPS-stimulated 16HBE. Figure S1E: Gene Ontology terms of trans-targeted genes of differentially expressed lncRNAs in LPS-stimulated 16HBE in biological process. Figure S1F: Gene Ontology terms of trans-targeted genes of differentially expressed lncRNAs in LPS-stimulated 16HBE in cellular component. Figure S1G: Gene Ontology terms of trans-targeted genes of differentially expressed lncRNAs in LPS-stimulated 16HBE in molecular function. Figure S1H: the KEGG enrichment analysis of trans-targeted genes of differentially expressed lncRNAs in LPS-stimulated 16HBE. Figure S1I: Gene Ontology terms of differentially expressed circRNAs in LPS-stimulated 16HBE in biological process. Figure S1J: Gene Ontology terms of differentially expressed miRNA in LPS-stimulated 16HBE in cellular component. Figure S1K: Gene Ontology terms of differentially expressed miRNA in LPS-stimulated 16HBE in molecular function. Figure S1L: the KEGG enrichment analysis of differentially expressed miRNA in LPS-stimulated 16HBE. [file 6831770.f1.zip › Figure S1A.pdf]

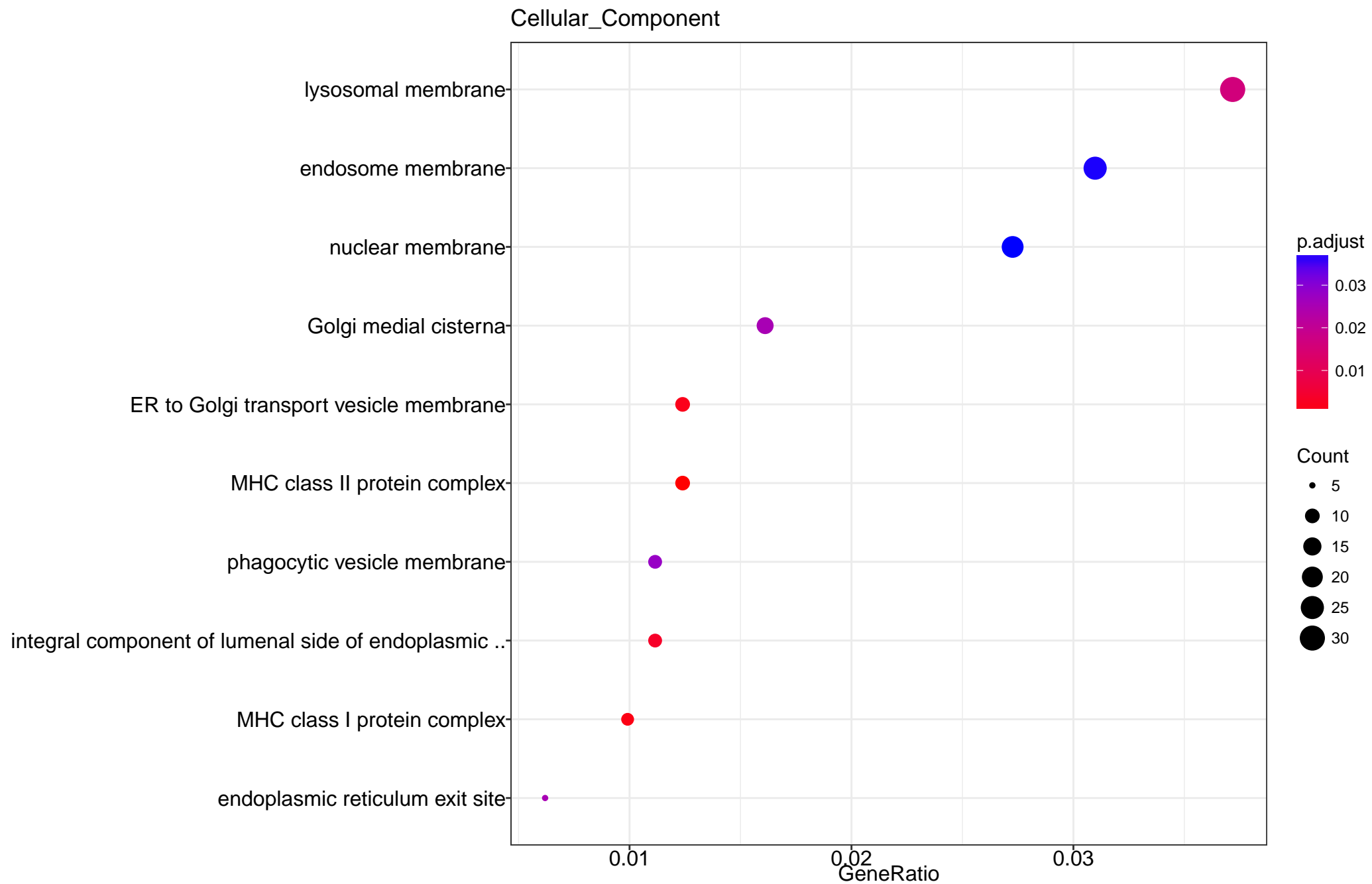

Supplement: Supplementary Materials — Figure S1A: Gene Ontology terms of cis-targeted genes of differentially expressed lncRNAs in LPS-stimulated 16HBE in biological process. Figure S1B: Gene Ontology terms of cis-targeted genes of differentially expressed lncRNAs in LPS-stimulated 16HBE in cellular component. Figure S1C: Gene Ontology terms of cis-targeted genes of differentially expressed lncRNAs in LPS-stimulated 16HBE in molecular function. Figure S1D: the KEGG enrichment analysis of cis-targeted genes of differentially expressed lncRNAs in LPS-stimulated 16HBE. Figure S1E: Gene Ontology terms of trans-targeted genes of differentially expressed lncRNAs in LPS-stimulated 16HBE in biological process. Figure S1F: Gene Ontology terms of trans-targeted genes of differentially expressed lncRNAs in LPS-stimulated 16HBE in cellular component. Figure S1G: Gene Ontology terms of trans-targeted genes of differentially expressed lncRNAs in LPS-stimulated 16HBE in molecular function. Figure S1H: the KEGG enrichment analysis of trans-targeted genes of differentially expressed lncRNAs in LPS-stimulated 16HBE. Figure S1I: Gene Ontology terms of differentially expressed circRNAs in LPS-stimulated 16HBE in biological process. Figure S1J: Gene Ontology terms of differentially expressed miRNA in LPS-stimulated 16HBE in cellular component. Figure S1K: Gene Ontology terms of differentially expressed miRNA in LPS-stimulated 16HBE in molecular function. Figure S1L: the KEGG enrichment analysis of differentially expressed miRNA in LPS-stimulated 16HBE. [file 6831770.f1.zip › Figure S1B.pdf]

## Molecular\_Function

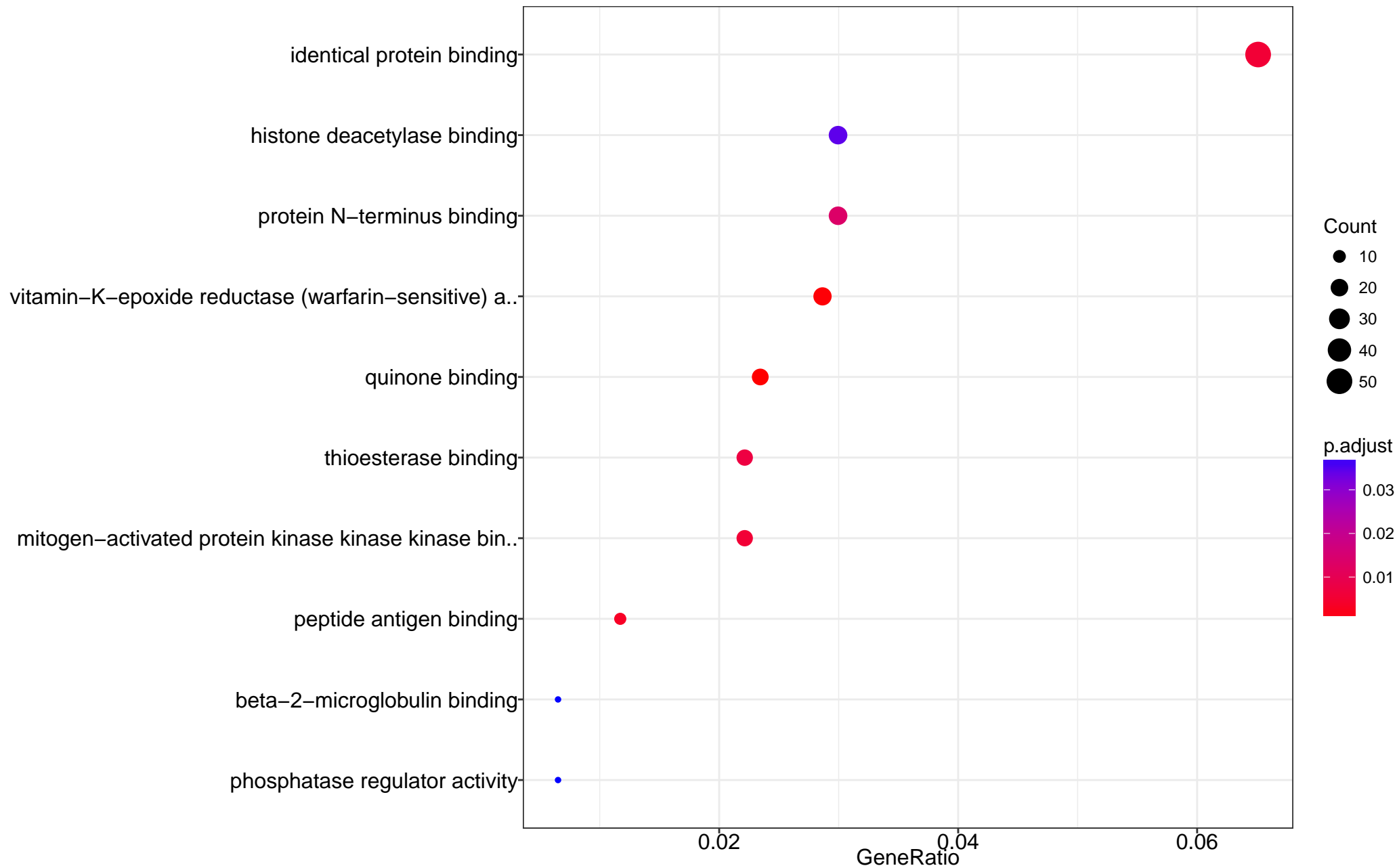

Supplement: Supplementary Materials — Figure S1A: Gene Ontology terms of cis-targeted genes of differentially expressed lncRNAs in LPS-stimulated 16HBE in biological process. Figure S1B: Gene Ontology terms of cis-targeted genes of differentially expressed lncRNAs in LPS-stimulated 16HBE in cellular component. Figure S1C: Gene Ontology terms of cis-targeted genes of differentially expressed lncRNAs in LPS-stimulated 16HBE in molecular function. Figure S1D: the KEGG enrichment analysis of cis-targeted genes of differentially expressed lncRNAs in LPS-stimulated 16HBE. Figure S1E: Gene Ontology terms of trans-targeted genes of differentially expressed lncRNAs in LPS-stimulated 16HBE in biological process. Figure S1F: Gene Ontology terms of trans-targeted genes of differentially expressed lncRNAs in LPS-stimulated 16HBE in cellular component. Figure S1G: Gene Ontology terms of trans-targeted genes of differentially expressed lncRNAs in LPS-stimulated 16HBE in molecular function. Figure S1H: the KEGG enrichment analysis of trans-targeted genes of differentially expressed lncRNAs in LPS-stimulated 16HBE. Figure S1I: Gene Ontology terms of differentially expressed circRNAs in LPS-stimulated 16HBE in biological process. Figure S1J: Gene Ontology terms of differentially expressed miRNA in LPS-stimulated 16HBE in cellular component. Figure S1K: Gene Ontology terms of differentially expressed miRNA in LPS-stimulated 16HBE in molecular function. Figure S1L: the KEGG enrichment analysis of differentially expressed miRNA in LPS-stimulated 16HBE. [file 6831770.f1.zip › Figure S1C.pdf]

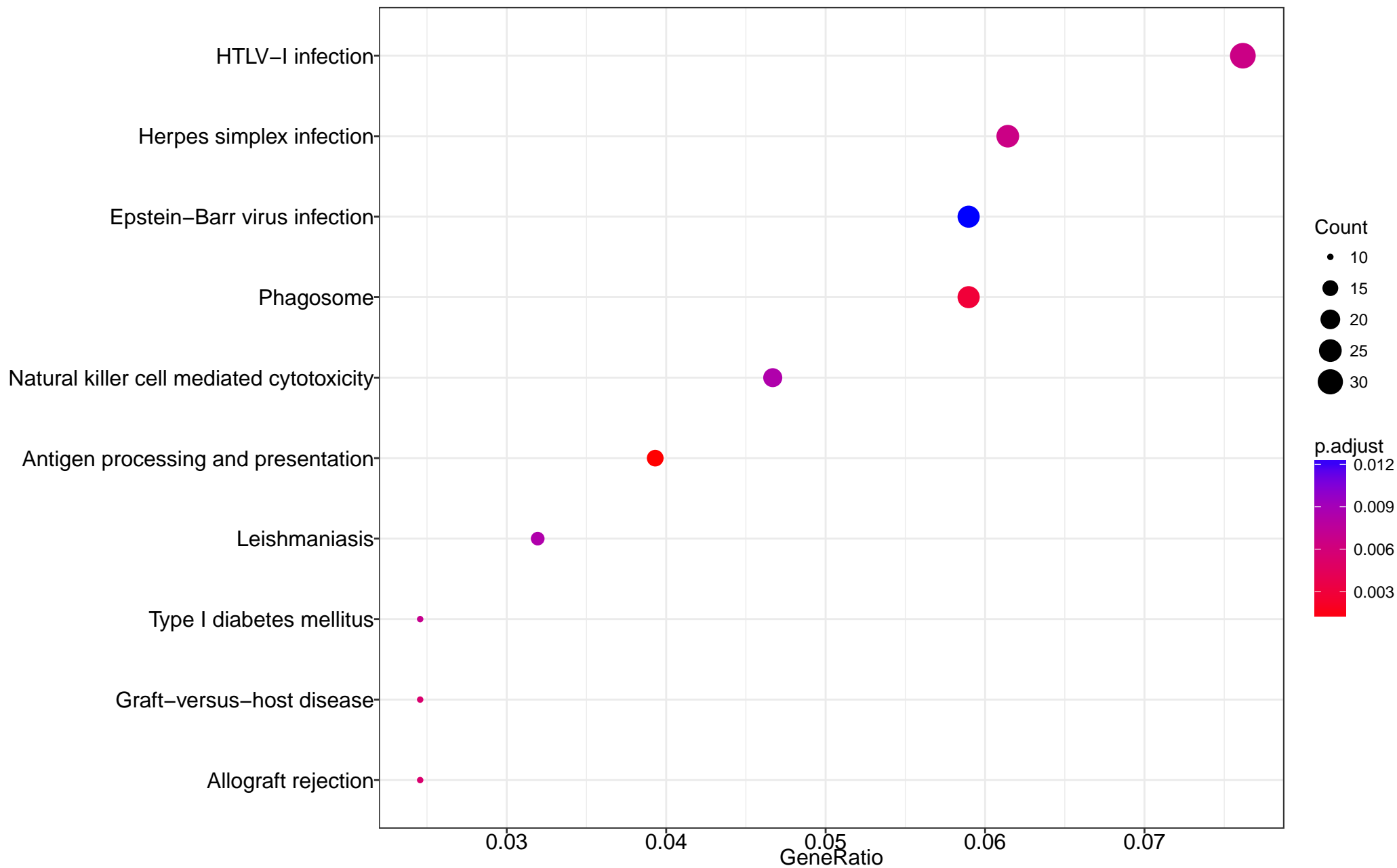

Supplement: Supplementary Materials — Figure S1A: Gene Ontology terms of cis-targeted genes of differentially expressed lncRNAs in LPS-stimulated 16HBE in biological process. Figure S1B: Gene Ontology terms of cis-targeted genes of differentially expressed lncRNAs in LPS-stimulated 16HBE in cellular component. Figure S1C: Gene Ontology terms of cis-targeted genes of differentially expressed lncRNAs in LPS-stimulated 16HBE in molecular function. Figure S1D: the KEGG enrichment analysis of cis-targeted genes of differentially expressed lncRNAs in LPS-stimulated 16HBE. Figure S1E: Gene Ontology terms of trans-targeted genes of differentially expressed lncRNAs in LPS-stimulated 16HBE in biological process. Figure S1F: Gene Ontology terms of trans-targeted genes of differentially expressed lncRNAs in LPS-stimulated 16HBE in cellular component. Figure S1G: Gene Ontology terms of trans-targeted genes of differentially expressed lncRNAs in LPS-stimulated 16HBE in molecular function. Figure S1H: the KEGG enrichment analysis of trans-targeted genes of differentially expressed lncRNAs in LPS-stimulated 16HBE. Figure S1I: Gene Ontology terms of differentially expressed circRNAs in LPS-stimulated 16HBE in biological process. Figure S1J: Gene Ontology terms of differentially expressed miRNA in LPS-stimulated 16HBE in cellular component. Figure S1K: Gene Ontology terms of differentially expressed miRNA in LPS-stimulated 16HBE in molecular function. Figure S1L: the KEGG enrichment analysis of differentially expressed miRNA in LPS-stimulated 16HBE. [file 6831770.f1.zip › Figure S1D.pdf]

# Biological\_Process

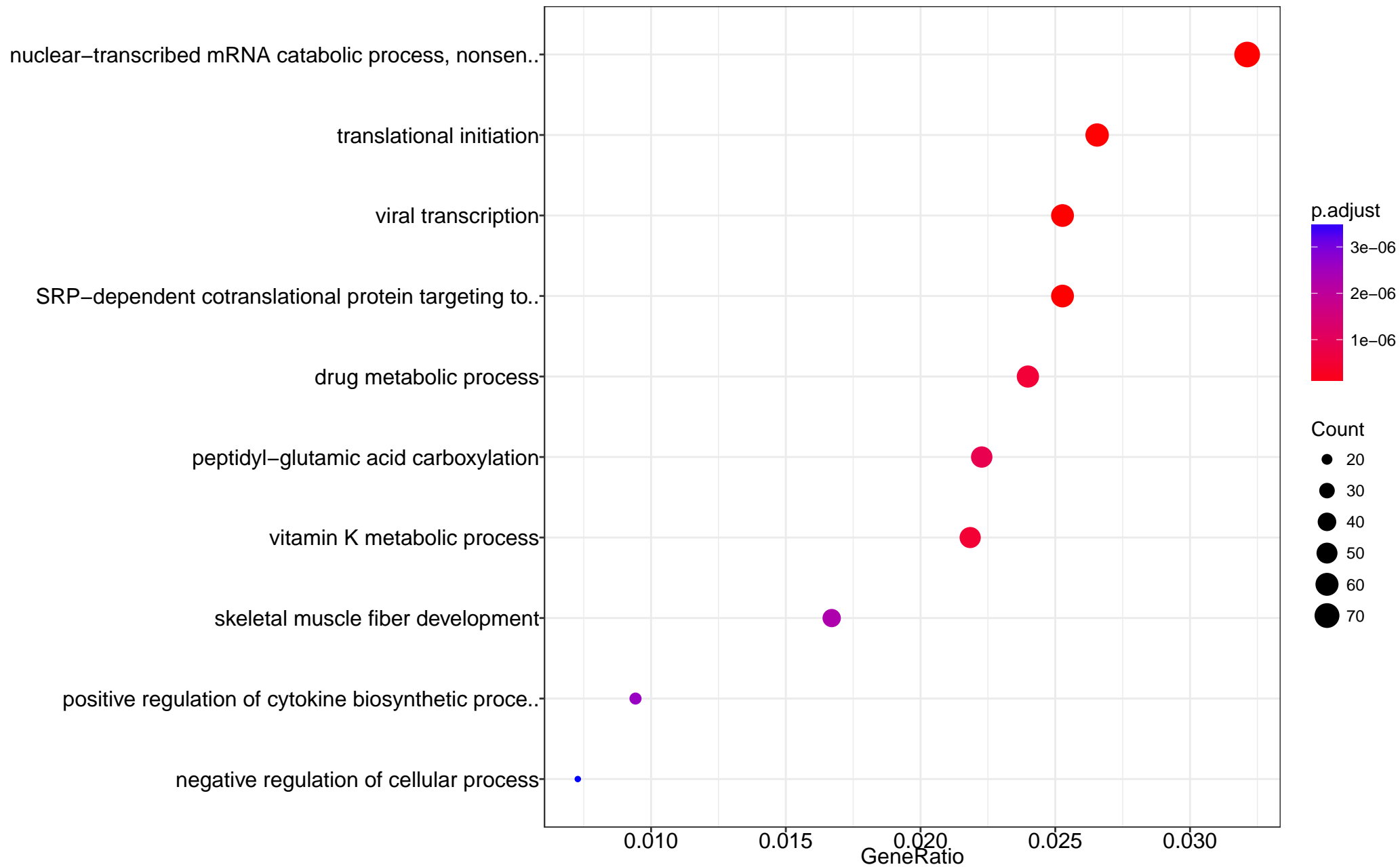

Supplement: Supplementary Materials — Figure S1A: Gene Ontology terms of cis-targeted genes of differentially expressed lncRNAs in LPS-stimulated 16HBE in biological process. Figure S1B: Gene Ontology terms of cis-targeted genes of differentially expressed lncRNAs in LPS-stimulated 16HBE in cellular component. Figure S1C: Gene Ontology terms of cis-targeted genes of differentially expressed lncRNAs in LPS-stimulated 16HBE in molecular function. Figure S1D: the KEGG enrichment analysis of cis-targeted genes of differentially expressed lncRNAs in LPS-stimulated 16HBE. Figure S1E: Gene Ontology terms of trans-targeted genes of differentially expressed lncRNAs in LPS-stimulated 16HBE in biological process. Figure S1F: Gene Ontology terms of trans-targeted genes of differentially expressed lncRNAs in LPS-stimulated 16HBE in cellular component. Figure S1G: Gene Ontology terms of trans-targeted genes of differentially expressed lncRNAs in LPS-stimulated 16HBE in molecular function. Figure S1H: the KEGG enrichment analysis of trans-targeted genes of differentially expressed lncRNAs in LPS-stimulated 16HBE. Figure S1I: Gene Ontology terms of differentially expressed circRNAs in LPS-stimulated 16HBE in biological process. Figure S1J: Gene Ontology terms of differentially expressed miRNA in LPS-stimulated 16HBE in cellular component. Figure S1K: Gene Ontology terms of differentially expressed miRNA in LPS-stimulated 16HBE in molecular function. Figure S1L: the KEGG enrichment analysis of differentially expressed miRNA in LPS-stimulated 16HBE. [file 6831770.f1.zip › Figure S1E.pdf]

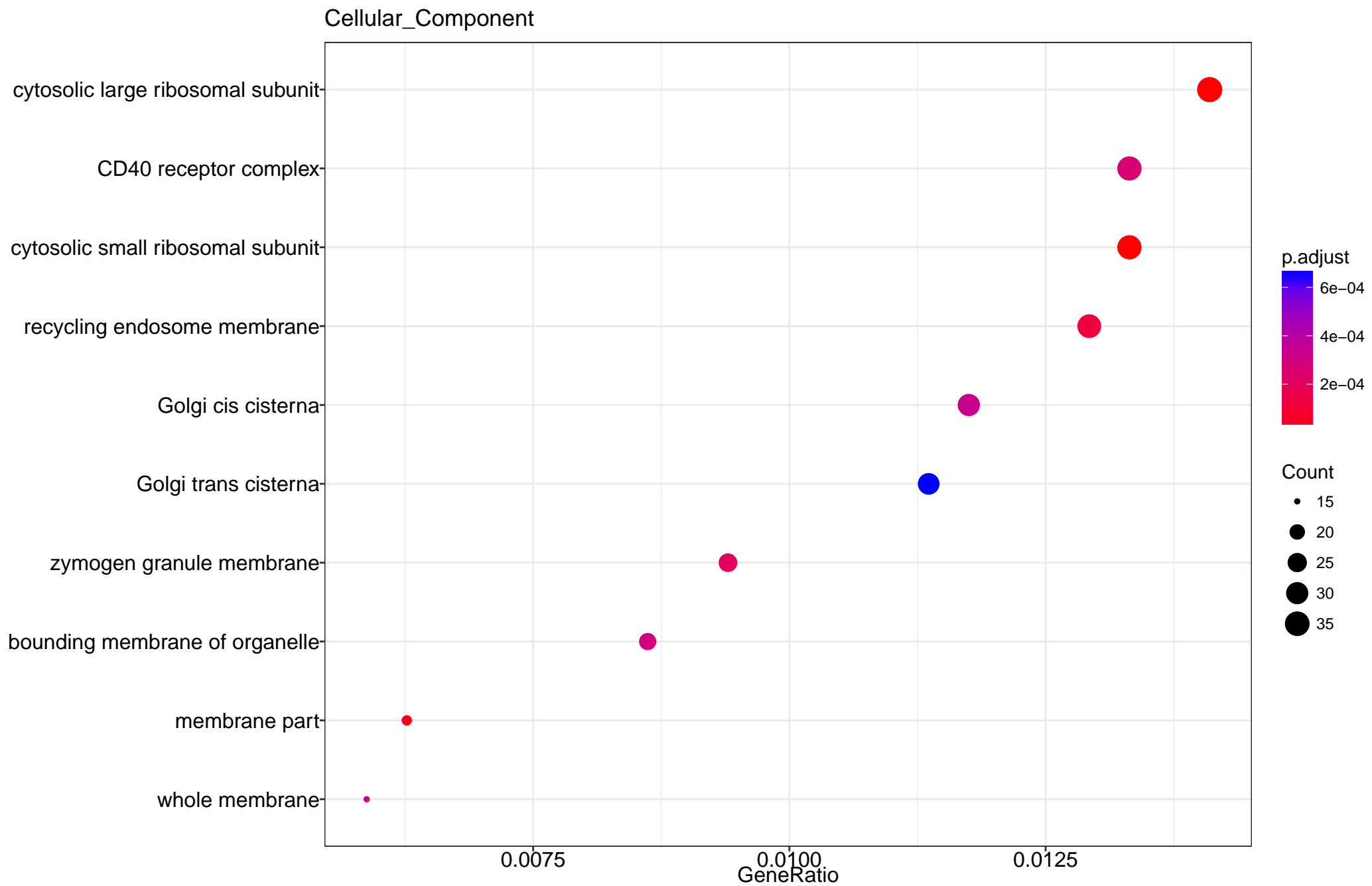

Supplement: Supplementary Materials — Figure S1A: Gene Ontology terms of cis-targeted genes of differentially expressed lncRNAs in LPS-stimulated 16HBE in biological process. Figure S1B: Gene Ontology terms of cis-targeted genes of differentially expressed lncRNAs in LPS-stimulated 16HBE in cellular component. Figure S1C: Gene Ontology terms of cis-targeted genes of differentially expressed lncRNAs in LPS-stimulated 16HBE in molecular function. Figure S1D: the KEGG enrichment analysis of cis-targeted genes of differentially expressed lncRNAs in LPS-stimulated 16HBE. Figure S1E: Gene Ontology terms of trans-targeted genes of differentially expressed lncRNAs in LPS-stimulated 16HBE in biological process. Figure S1F: Gene Ontology terms of trans-targeted genes of differentially expressed lncRNAs in LPS-stimulated 16HBE in cellular component. Figure S1G: Gene Ontology terms of trans-targeted genes of differentially expressed lncRNAs in LPS-stimulated 16HBE in molecular function. Figure S1H: the KEGG enrichment analysis of trans-targeted genes of differentially expressed lncRNAs in LPS-stimulated 16HBE. Figure S1I: Gene Ontology terms of differentially expressed circRNAs in LPS-stimulated 16HBE in biological process. Figure S1J: Gene Ontology terms of differentially expressed miRNA in LPS-stimulated 16HBE in cellular component. Figure S1K: Gene Ontology terms of differentially expressed miRNA in LPS-stimulated 16HBE in molecular function. Figure S1L: the KEGG enrichment analysis of differentially expressed miRNA in LPS-stimulated 16HBE. [file 6831770.f1.zip › Figure S1F.pdf]

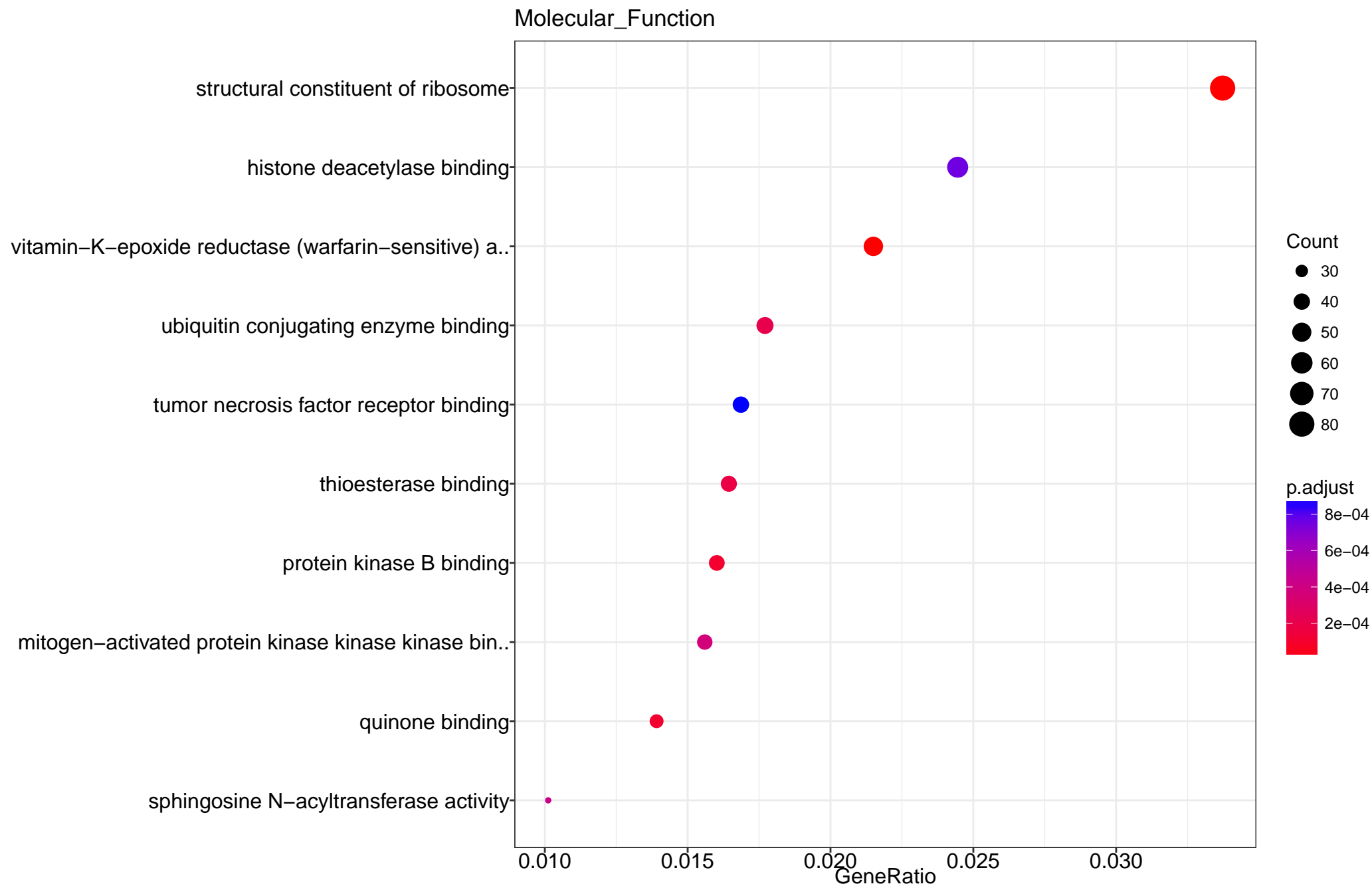

Supplement: Supplementary Materials — Figure S1A: Gene Ontology terms of cis-targeted genes of differentially expressed lncRNAs in LPS-stimulated 16HBE in biological process. Figure S1B: Gene Ontology terms of cis-targeted genes of differentially expressed lncRNAs in LPS-stimulated 16HBE in cellular component. Figure S1C: Gene Ontology terms of cis-targeted genes of differentially expressed lncRNAs in LPS-stimulated 16HBE in molecular function. Figure S1D: the KEGG enrichment analysis of cis-targeted genes of differentially expressed lncRNAs in LPS-stimulated 16HBE. Figure S1E: Gene Ontology terms of trans-targeted genes of differentially expressed lncRNAs in LPS-stimulated 16HBE in biological process. Figure S1F: Gene Ontology terms of trans-targeted genes of differentially expressed lncRNAs in LPS-stimulated 16HBE in cellular component. Figure S1G: Gene Ontology terms of trans-targeted genes of differentially expressed lncRNAs in LPS-stimulated 16HBE in molecular function. Figure S1H: the KEGG enrichment analysis of trans-targeted genes of differentially expressed lncRNAs in LPS-stimulated 16HBE. Figure S1I: Gene Ontology terms of differentially expressed circRNAs in LPS-stimulated 16HBE in biological process. Figure S1J: Gene Ontology terms of differentially expressed miRNA in LPS-stimulated 16HBE in cellular component. Figure S1K: Gene Ontology terms of differentially expressed miRNA in LPS-stimulated 16HBE in molecular function. Figure S1L: the KEGG enrichment analysis of differentially expressed miRNA in LPS-stimulated 16HBE. [file 6831770.f1.zip › Figure S1G.pdf]

KEGG\_pathway

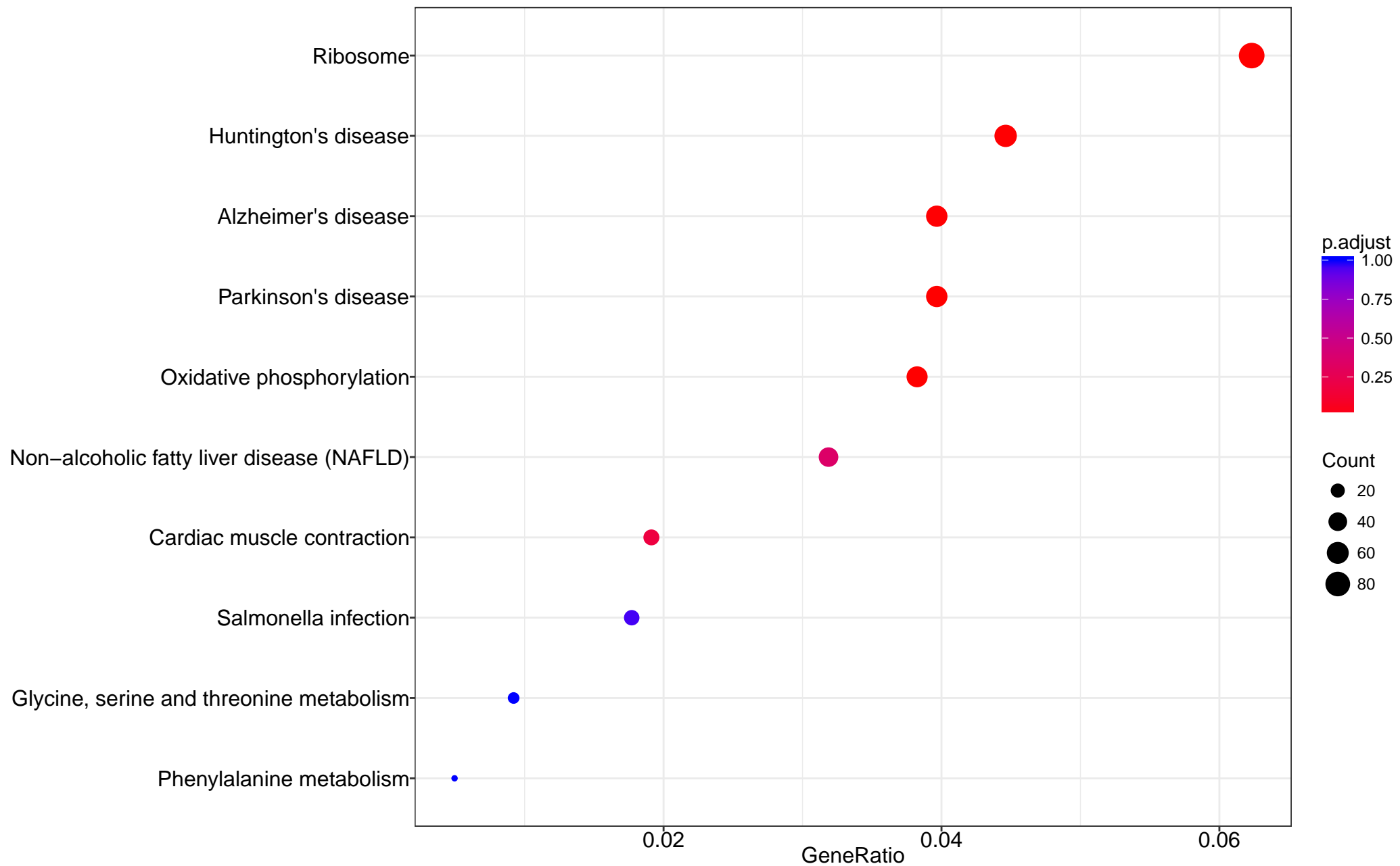

Supplement: Supplementary Materials — Figure S1A: Gene Ontology terms of cis-targeted genes of differentially expressed lncRNAs in LPS-stimulated 16HBE in biological process. Figure S1B: Gene Ontology terms of cis-targeted genes of differentially expressed lncRNAs in LPS-stimulated 16HBE in cellular component. Figure S1C: Gene Ontology terms of cis-targeted genes of differentially expressed lncRNAs in LPS-stimulated 16HBE in molecular function. Figure S1D: the KEGG enrichment analysis of cis-targeted genes of differentially expressed lncRNAs in LPS-stimulated 16HBE. Figure S1E: Gene Ontology terms of trans-targeted genes of differentially expressed lncRNAs in LPS-stimulated 16HBE in biological process. Figure S1F: Gene Ontology terms of trans-targeted genes of differentially expressed lncRNAs in LPS-stimulated 16HBE in cellular component. Figure S1G: Gene Ontology terms of trans-targeted genes of differentially expressed lncRNAs in LPS-stimulated 16HBE in molecular function. Figure S1H: the KEGG enrichment analysis of trans-targeted genes of differentially expressed lncRNAs in LPS-stimulated 16HBE. Figure S1I: Gene Ontology terms of differentially expressed circRNAs in LPS-stimulated 16HBE in biological process. Figure S1J: Gene Ontology terms of differentially expressed miRNA in LPS-stimulated 16HBE in cellular component. Figure S1K: Gene Ontology terms of differentially expressed miRNA in LPS-stimulated 16HBE in molecular function. Figure S1L: the KEGG enrichment analysis of differentially expressed miRNA in LPS-stimulated 16HBE. [file 6831770.f1.zip › Figure S1H.pdf]

# Biological\_Process

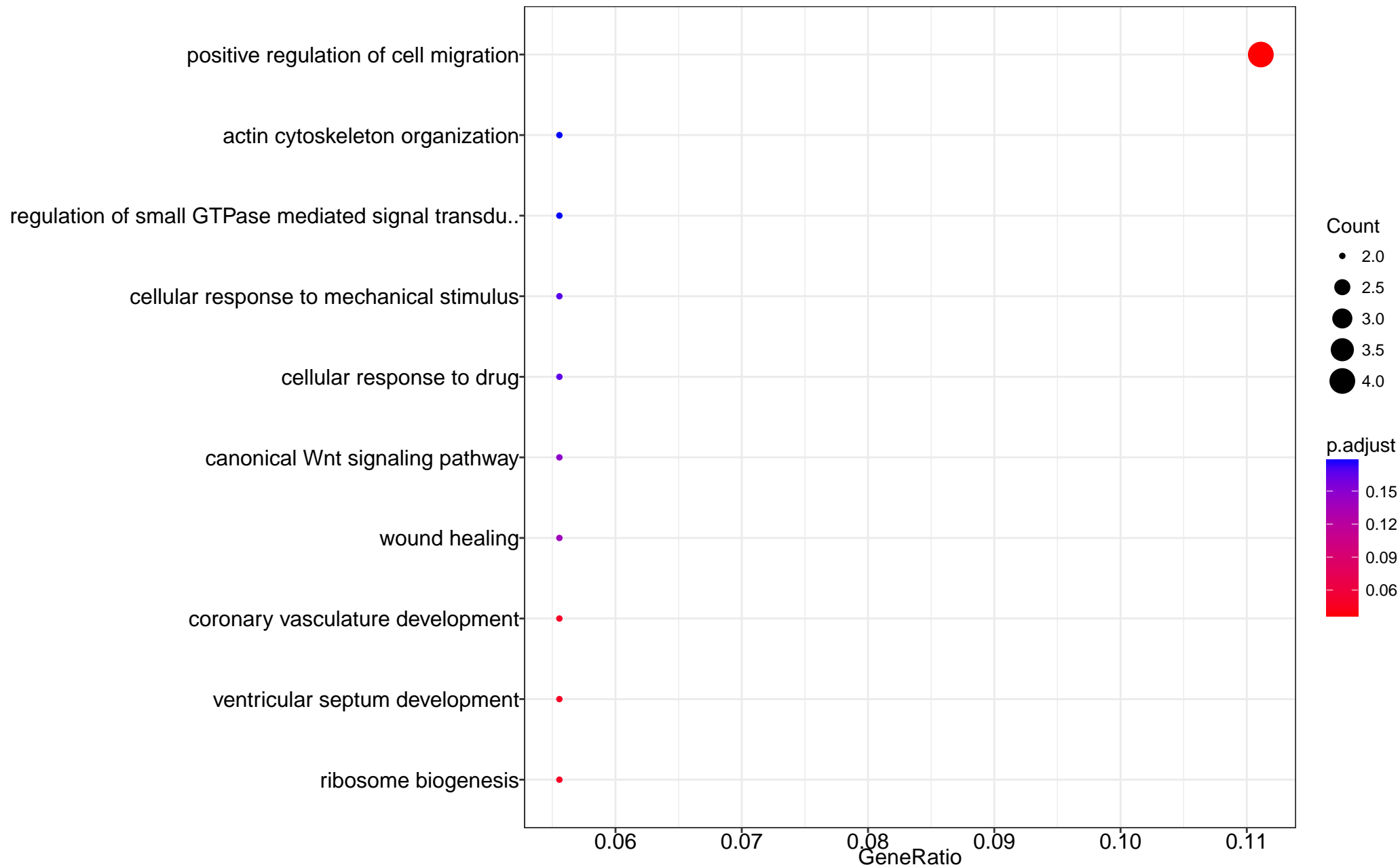

Supplement: Supplementary Materials — Figure S1A: Gene Ontology terms of cis-targeted genes of differentially expressed lncRNAs in LPS-stimulated 16HBE in biological process. Figure S1B: Gene Ontology terms of cis-targeted genes of differentially expressed lncRNAs in LPS-stimulated 16HBE in cellular component. Figure S1C: Gene Ontology terms of cis-targeted genes of differentially expressed lncRNAs in LPS-stimulated 16HBE in molecular function. Figure S1D: the KEGG enrichment analysis of cis-targeted genes of differentially expressed lncRNAs in LPS-stimulated 16HBE. Figure S1E: Gene Ontology terms of trans-targeted genes of differentially expressed lncRNAs in LPS-stimulated 16HBE in biological process. Figure S1F: Gene Ontology terms of trans-targeted genes of differentially expressed lncRNAs in LPS-stimulated 16HBE in cellular component. Figure S1G: Gene Ontology terms of trans-targeted genes of differentially expressed lncRNAs in LPS-stimulated 16HBE in molecular function. Figure S1H: the KEGG enrichment analysis of trans-targeted genes of differentially expressed lncRNAs in LPS-stimulated 16HBE. Figure S1I: Gene Ontology terms of differentially expressed circRNAs in LPS-stimulated 16HBE in biological process. Figure S1J: Gene Ontology terms of differentially expressed miRNA in LPS-stimulated 16HBE in cellular component. Figure S1K: Gene Ontology terms of differentially expressed miRNA in LPS-stimulated 16HBE in molecular function. Figure S1L: the KEGG enrichment analysis of differentially expressed miRNA in LPS-stimulated 16HBE. [file 6831770.f1.zip › Figure S1I.pdf]

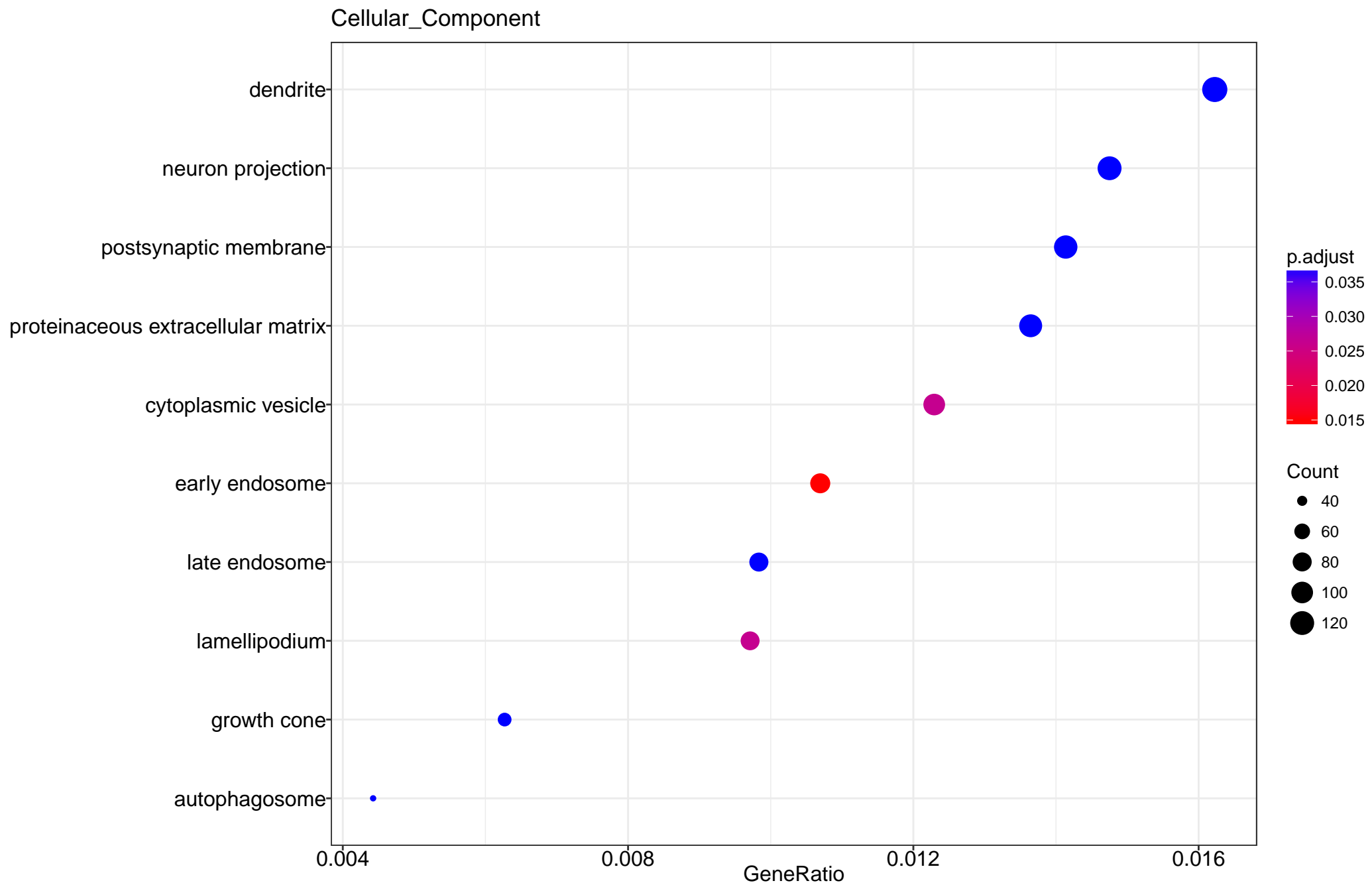

Supplement: Supplementary Materials — Figure S1A: Gene Ontology terms of cis-targeted genes of differentially expressed lncRNAs in LPS-stimulated 16HBE in biological process. Figure S1B: Gene Ontology terms of cis-targeted genes of differentially expressed lncRNAs in LPS-stimulated 16HBE in cellular component. Figure S1C: Gene Ontology terms of cis-targeted genes of differentially expressed lncRNAs in LPS-stimulated 16HBE in molecular function. Figure S1D: the KEGG enrichment analysis of cis-targeted genes of differentially expressed lncRNAs in LPS-stimulated 16HBE. Figure S1E: Gene Ontology terms of trans-targeted genes of differentially expressed lncRNAs in LPS-stimulated 16HBE in biological process. Figure S1F: Gene Ontology terms of trans-targeted genes of differentially expressed lncRNAs in LPS-stimulated 16HBE in cellular component. Figure S1G: Gene Ontology terms of trans-targeted genes of differentially expressed lncRNAs in LPS-stimulated 16HBE in molecular function. Figure S1H: the KEGG enrichment analysis of trans-targeted genes of differentially expressed lncRNAs in LPS-stimulated 16HBE. Figure S1I: Gene Ontology terms of differentially expressed circRNAs in LPS-stimulated 16HBE in biological process. Figure S1J: Gene Ontology terms of differentially expressed miRNA in LPS-stimulated 16HBE in cellular component. Figure S1K: Gene Ontology terms of differentially expressed miRNA in LPS-stimulated 16HBE in molecular function. Figure S1L: the KEGG enrichment analysis of differentially expressed miRNA in LPS-stimulated 16HBE. [file 6831770.f1.zip › Figure S1J.pdf]

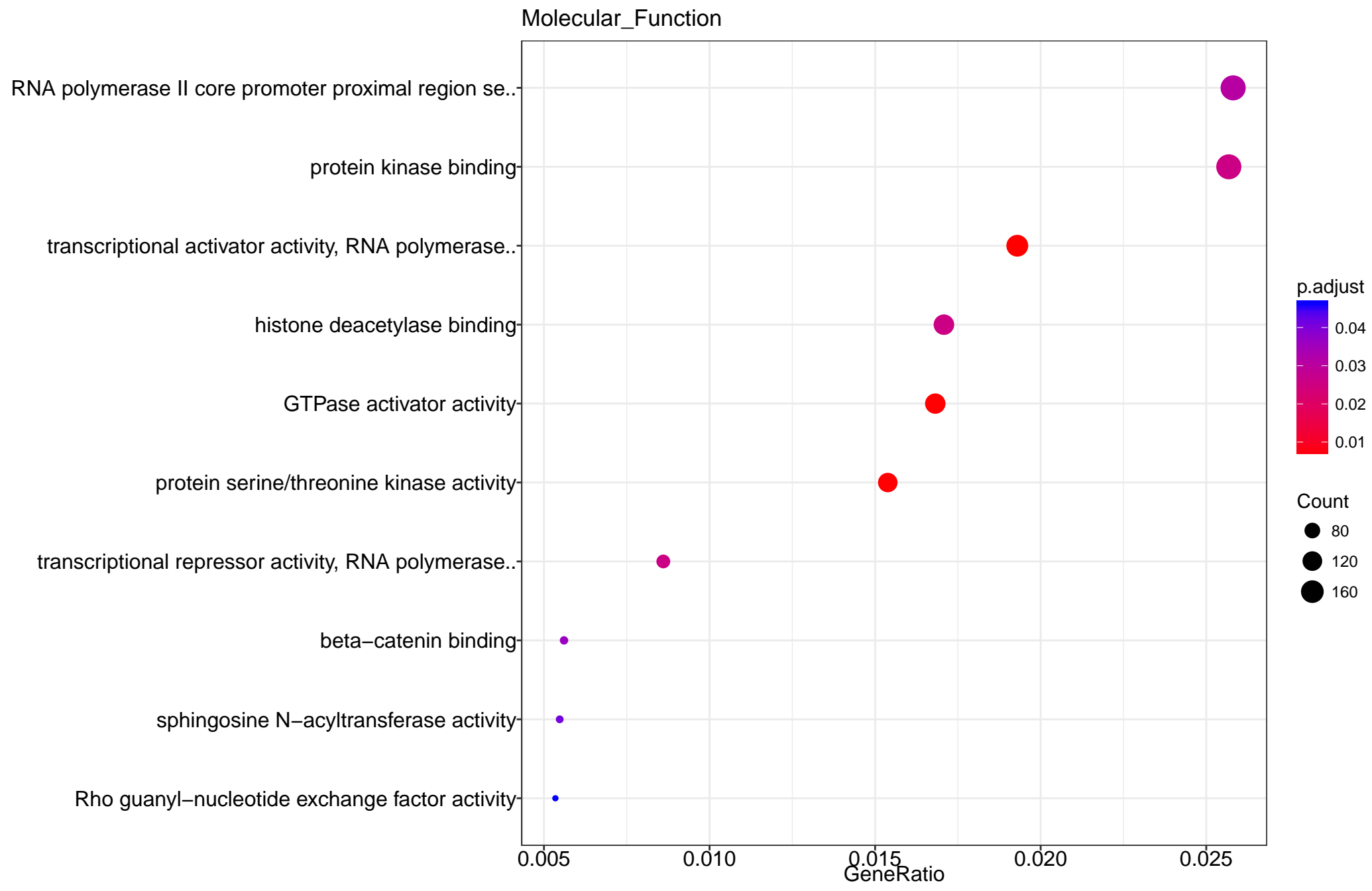

Supplement: Supplementary Materials — Figure S1A: Gene Ontology terms of cis-targeted genes of differentially expressed lncRNAs in LPS-stimulated 16HBE in biological process. Figure S1B: Gene Ontology terms of cis-targeted genes of differentially expressed lncRNAs in LPS-stimulated 16HBE in cellular component. Figure S1C: Gene Ontology terms of cis-targeted genes of differentially expressed lncRNAs in LPS-stimulated 16HBE in molecular function. Figure S1D: the KEGG enrichment analysis of cis-targeted genes of differentially expressed lncRNAs in LPS-stimulated 16HBE. Figure S1E: Gene Ontology terms of trans-targeted genes of differentially expressed lncRNAs in LPS-stimulated 16HBE in biological process. Figure S1F: Gene Ontology terms of trans-targeted genes of differentially expressed lncRNAs in LPS-stimulated 16HBE in cellular component. Figure S1G: Gene Ontology terms of trans-targeted genes of differentially expressed lncRNAs in LPS-stimulated 16HBE in molecular function. Figure S1H: the KEGG enrichment analysis of trans-targeted genes of differentially expressed lncRNAs in LPS-stimulated 16HBE. Figure S1I: Gene Ontology terms of differentially expressed circRNAs in LPS-stimulated 16HBE in biological process. Figure S1J: Gene Ontology terms of differentially expressed miRNA in LPS-stimulated 16HBE in cellular component. Figure S1K: Gene Ontology terms of differentially expressed miRNA in LPS-stimulated 16HBE in molecular function. Figure S1L: the KEGG enrichment analysis of differentially expressed miRNA in LPS-stimulated 16HBE. [file 6831770.f1.zip › Figure S1K.pdf]

# KEGG\_pathway

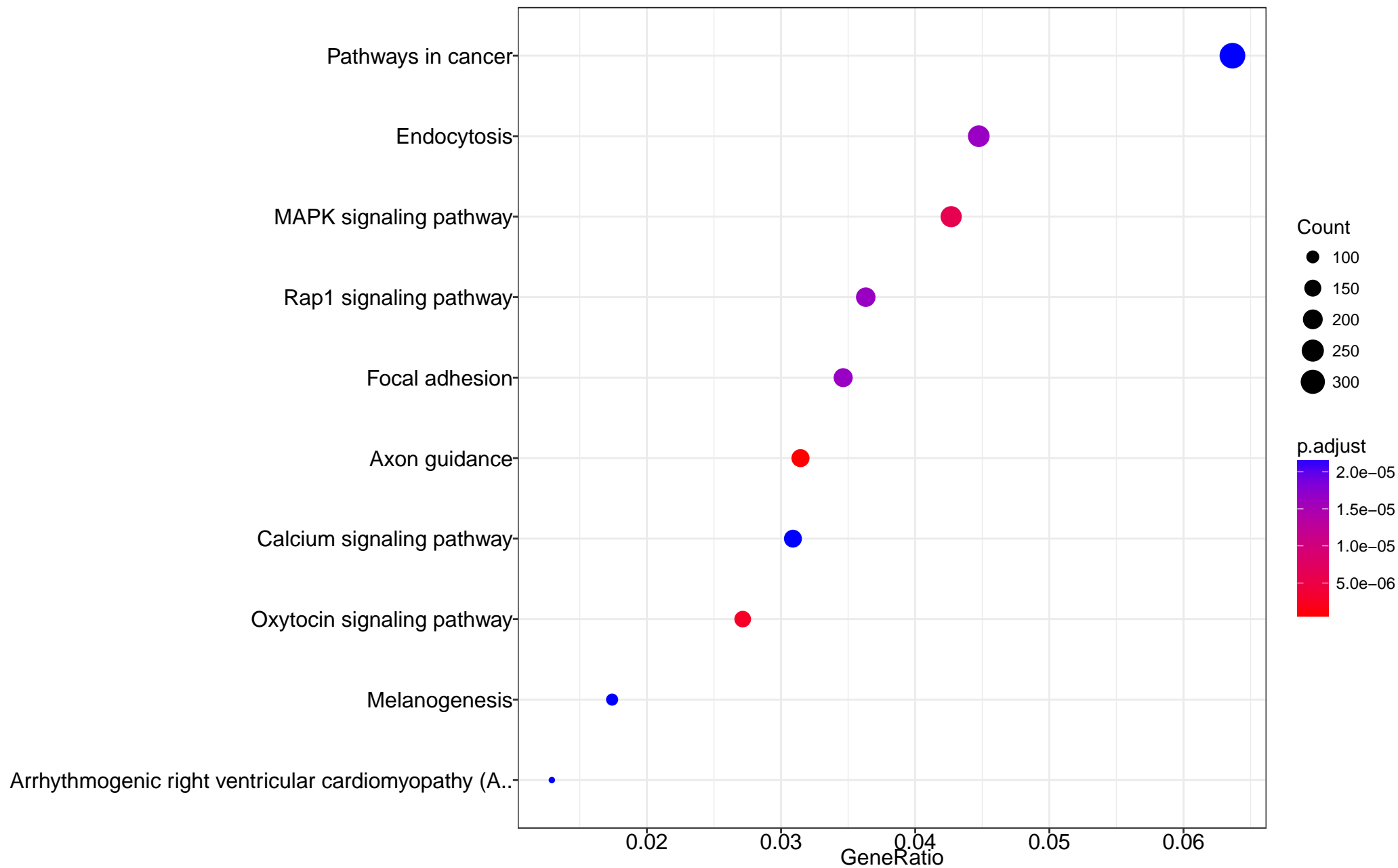

Supplement: Supplementary Materials — Figure S1A: Gene Ontology terms of cis-targeted genes of differentially expressed lncRNAs in LPS-stimulated 16HBE in biological process. Figure S1B: Gene Ontology terms of cis-targeted genes of differentially expressed lncRNAs in LPS-stimulated 16HBE in cellular component. Figure S1C: Gene Ontology terms of cis-targeted genes of differentially expressed lncRNAs in LPS-stimulated 16HBE in molecular function. Figure S1D: the KEGG enrichment analysis of cis-targeted genes of differentially expressed lncRNAs in LPS-stimulated 16HBE. Figure S1E: Gene Ontology terms of trans-targeted genes of differentially expressed lncRNAs in LPS-stimulated 16HBE in biological process. Figure S1F: Gene Ontology terms of trans-targeted genes of differentially expressed lncRNAs in LPS-stimulated 16HBE in cellular component. Figure S1G: Gene Ontology terms of trans-targeted genes of differentially expressed lncRNAs in LPS-stimulated 16HBE in molecular function. Figure S1H: the KEGG enrichment analysis of trans-targeted genes of differentially expressed lncRNAs in LPS-stimulated 16HBE. Figure S1I: Gene Ontology terms of differentially expressed circRNAs in LPS-stimulated 16HBE in biological process. Figure S1J: Gene Ontology terms of differentially expressed miRNA in LPS-stimulated 16HBE in cellular component. Figure S1K: Gene Ontology terms of differentially expressed miRNA in LPS-stimulated 16HBE in molecular function. Figure S1L: the KEGG enrichment analysis of differentially expressed miRNA in LPS-stimulated 16HBE. [file 6831770.f1.zip › Figure S1L.pdf]
